# New Species of *Trichoderma* Isolated as Endophytes and Saprobes from Southwest China

**DOI:** 10.3390/jof7060467

**Published:** 2021-06-09

**Authors:** Hua Zheng, Min Qiao, Yifan Lv, Xing Du, Ke-Qin Zhang, Zefen Yu

**Affiliations:** 1 Laboratory for Conservation and Utilization of Bio-Resources, Key Laboratory for Microbial Resources of the Ministry of Education, Yunnan University, Kunming 650091, Yunnan, China; zhenghua@mail.ynu.edu.cn (H.Z.); qiaoming@ynu.edu.cn (M.Q.); LV19950131@hotmail.com (Y.L.); duxing@angelyeast.com (X.D.); kqzhang@ynu.edu.cn (K.-Q.Z.); 2 School of Life Sciences, Yunnan University, Kunming 650091, Yunnan, China

**Keywords:** soil-inhabiting, aquatic, endophytic, Hypocreales, new species, *Trichoderma*

## Abstract

During the investigation of endophytic fungi diversity in aquatic plants and the fungal diversity in soil in southwest China, we obtained 208 isolates belonging to *Trichoderma*, including 28 isolates as endophytes from aquatic plants and 180 isolates as saprobes from soil, respectively. Finally, 23 new species of *Trichoderma* are recognized by further studies. Their phylogenetic positions are determined by sequence analyses of the combined partial sequences of translation elongation factor 1-alpha (*tef*1) and gene encoding of the second largest nuclear RNA polymerase subunit (*rpb*2). The results revealed that the 23 new species are distributed in nine known clades. The morphology and culture characteristics are observed, described and illustrated in detail. Distinctions between the new species and their close relatives were compared and discussed. These include: *Trichoderma achlamydosporum*, *T. amoenum*, *T. anaharzianum*, *T. anisohamatum*, *T. aquatica*, *T. asiaticum*, *T. asymmetricum*, *T. inaequilaterale*, *T. inconspicuum*, *T. insigne*, *T. obovatum*, *T. paraviride*, *T. pluripenicillatum*, *T. propepolypori*, *T. pseudoasiaticum*, *T. pseudoasperelloides*, *T. scorpioideum*, *T. simile*, *T. subazureum*, *T. subuliforme*, *T. supraverticillatum*, *T. tibetica*, and *T. uncinatum.*

## 1. Introduction

*Trichoderma* Pers, the anamorphic state of *Hypocrea* Fr. (Ascomycota, Sordariomycetes, Hypocreales), is an ecologically and economically important genus. According to the International Code of Nomenclature for algae, fungi, and plants [1], pleomorphic individuals no longer have more than one name, *Trichoderma* is now the legal name and *Hypocrea* is considered synonym [2]. Members of *Trichoderma* are widely distributed, having been found in various ecosystems, such as soil, decaying wood, plant leaves, bark and root systems, and also living as endophytes in plant tissues [3,4,5,6,7,8,9]. Many *Trichoderma* species have been used or encountered in many human activities, including as biocontrol agents of plant diseases, promoters of plant growth, enhancers of soil fertility in agriculture, producers of enzymes and antibiotics, processors of food, and handlers of pulp in industry [3,10,11]. In addition, they have great potential in soil and water pollution remediation, e.g., *T. viride* Pers. and *T. atroviride* P. Karst. are good bioremediators for some heavy metal ions [12,13]. Some *Trichoderma* species can also be used to manufacture gold or silver nanoparticles in nanotechnology [14,15]. However, the members of some species of the genus *Trichoderma* are the causal agents of diseases in commercially produced mushrooms, resulting in serious losses in mushroom production [16,17].

The genus *Trichoderma*, typified with *T. viride* Pers., was proposed as a genus by Persoon in 1794 [18], and originally included three other species based on the different colors of conidia. It has been proven that only *T. viride* remained in the genus. In 1871, Harz proposed the first accurate definition of *Trichoderma*, and emphasized the importance of microscopic characteristics, especially phialide. Subsequently, the first taxonomy system of *Trichoderma* species was put forward by Rifai in 1969, divided *Trichoderma* into nine species aggregates, mainly according to the morphological characteristics of conidiophores and phialides [19]. Since then, the number of *Trichoderma* species has increased dramatically. Currently, there are more than 340 species recognized in the genus (Index Fungorum, May 2021).

Most *Trichoderma* species were isolated as saprobes from soil. In comparison, there are also some *Trichoderma* species found as endophytes or isolated from aquatic ecosystems. Of these *Trichoderma* species, which isolated as endophytes, most belong to the *Harzianum*, *Koningiopsis*, and *Hamatum* clades. Previous studies investigated some *Trichoderma* species living as endophytes and found that *Trichoderma* species as innoxious endosymbionts were abundant in woody plants stems (vascular cambium and phloem), such as *Cola* spp., *Herrania* spp., *Hevea* spp., *Theobroma* spp. [20,21,22,23,24,25]. *Trichoderma* species in aquatic environment also showed biodiversity, especially in marine habitats. For example, Paz et al. [26] reported 10 *Trichoderma* species associated with Mediterranean sponges and Gal-Hemed et al. [27] isolated 29 *Trichoderma* strains from Mediterranean *Psammocinia* sp. sponges. However, endophytic *Trichoderma* species from freshwater ecosystems have not been reported.

Traditionally, taxonomic studies of members of the genus *Trichoderma* were based on morphology. However, as more and more novel species have been discovered, it has been difficult to distinguish them only by means of morphological observation because species in this genus are highly similar in morphology. For instance, most species in this genus usually grow fast, produce highly branched conidiophores, cylindrical to nearly subglobose phialides and ellipsoidal and globose conidia. Moreover, the morphological characters may change with different environmental conditions [28]. Therefore, the use of DNA sequence analysis became a new method in fungal phylogenetics and systematics. Of these, multi-locus molecular phylogeny enables the rapid and accurate identification of *Trichoderma* species, and a significant number of *Trichoderma* species have been reported based on molecular phylogenetic evidence [29,30,31,32,33].

China has enormous fungal diversity, with the Southwestern region in China assessed as one of the world’s 34 biodiversity hotspots [34]. In recent years, we investigated the endophytic fungi diversity in aquatic plants in southwestern China, and obtained over 2000 isolates. After preliminary examination and classification by ITS (the internal transcribed spacer region) barcoding, 28 isolates were found to belong to *Trichoderma*. Furthermore, we also surveyed the fungal diversity in soil in Yunnan Province, and isolated 180 isolates belonging to *Trichoderma* species. Based on the multi-locus phylogenetic analysis and morphological features for all 208 isolates, 23 new species were recognized within the genus *Trichoderma*. This study significantly expands the worldwide diversity of *Trichoderma* and provides descriptions of new taxa.

## 2. Materials and Methods

### 2.1. Sample Collection

Soil samples were mainly collected from Yunnan Province, China, and placed into sterile, self-sealing plastic bags. Aquatic plants were collected from lakes, rivers, ponds, reservoirs and wetlands in provinces of Yunnan, Guizhou, Sichuan, and Tibet Autonomous Region. These plant samples were also placed into sterile self-sealing plastic bags. All the samples were transported to the lab and stored at 4 °C until processing.

### 2.2. Isolation of Fungi

For soil samples, soil fungi were isolated by gradient dilution and the spread plate method [35]. Three dilutions (10^−1^, 10^−2^, and 10^−3^) were prepared with 10 g soil and 90 mL sterile water, 0.2 mL of each dilution was spread on a 90 mm Rose Bengal Agar (RBA) plates (Guangdong Huankai Microbial Sci and Tech, Guangzhou, Guangdong Province, China), supplemented with two antibiotics (0.5 g l^−1^ penicillin G and 0.5 g l^−1^ streptomycin) [36]. Then, these plates were cultured at 25 °C. After 3–5 days, single colonies were isolated into pure culture and grown on potato dextrose agar plates (PDA; 200 g potato, 20 g glucose, 18 g agar, 1 L distilled water). For plant samples, endophytic fungi were isolated by incubating surface-disinfected tissue segments (5 mm diam.) on RBA plates according to the method described by Zheng et al. [37]. These Petri dishes were sealed, incubated at 25 °C, and examined periodically. When fungi grew out from the tissue segment, a few hyphal fragments were picked up and transferred to PDA plates. 

The pure cultures and dried cultures were deposited in the Herbarium of the Laboratory for Conservation and Utilization of Bio-Resources, Yunnan University, Kunming, Yunnan, China (YMF), the China Center for type Culture Collection (CCTCC), and the China General Microbiological Culture Collection Center (CGMCC). 

### 2.3. Growth Rate Determination and Morphology

Growth rates were determined on 9-cm-diameter Petri dishes of PDA, cornmeal dextrose agar (CMD; 40 g cornmeal, 20 g glucose, 18 g agar, 1 L distilled water) and synthetic low nutrient agar (SNA; 1 g KH_2_PO_4_, 1 g KNO_3_, 0.5 g MgSO_4_, 0.5 g KCl, 0.2 g glucose, 0.2 g sucrose, 18 g agar, 1 L distilled water) at 25, 30, and 35 °C under alternating 12 h light and 12 h darkness [38]. Colony diameters were measured after 3 days and the time when mycelia entirely covered the surface of plate was also recorded. Furthermore, the morphological characters of colonies, such as colony appearance, color, and spore production, were recorded at the same time. For microscopic morphology, photographs were taken with an Olympus BX51 microscope (Tokyo, Japan) connected to a DP controller digital camera. Microscopic characteristics were made from cultures growing on CMD or SNA at 25 °C. Colonies were photographed after 7 days and conidia were photographed after 14 days of incubation.

### 2.4. DNA Extraction, PCR Amplification and Sequencing

DNA was extracted from fresh mycelium harvested from PDA plates after 4 days, as described by Turner et al [39]. Fragments of the internal transcribed spacers (ITS), RNA Polymerase II subunit B (*rpb*2), and translation elongation factor 1-alpha (*tef*1) were amplified with the following primer pairs: ITS4 and ITS5 for ITS [40], EF1–728F [41] and TEF1LLErev [42] for *tef*1, and fRPB2–5f and fRPB2–7cr for *rpb*2 [43], respectively. Each 25 μL PCR reaction volume consisted of 12.5 μL T5 Super PCR Mix (containing Taq polymerase, dNTP and Mg^2+^, Beijing TsingKe Biotech Co., Ltd., Beijing, China), 1 μL of forward primer (10 µM), 1 μL of reverse primer (10 µM), 1μL DNA template, 5 μL of PCR buffer and 4.5 μL sterile water. PCR reactions were run in an Eppendorf Mastercycler (Eppendorf, Hamburg, Germany) following the PCR thermal cycle programs described by Chen & Zhuang [44]. PCR products were purified by using the PCR product purification kit (Biocolor BioScience and Technology Co., Shanghai, China), and forward and reverse sequenced on an ABI 3730 XL DNA sequencer (Applied Biosystems, Foster City, CA, USA) with the same primers, using a Thermo Sequenase Kit as described by Kindermann et al. [45]. These sequences were deposited in the GenBank database at the National Center for Biotechnology Information (NCBI) and the accession numbers are listed in Supplementary Table S1.

### 2.5. Phylogenetic Analyses

The phylogeny was reconstructed with sequences of *rpb*2 and *tef*1. Most of the sequences analyzed here were obtained from GenBank based on previous publications [6,44,46], and the remaining sequences were obtained by BLAST searches. Both the reference sequences and new generated sequences in this study are listed in Supplementary Table S1. DNA sequence data of *rpb*2 and *tef*1 were aligned using Clustal X 1.83 [47] with the default parameters, then the consensus sequences were manually adjusted and linked through BioEdit v.7.0 [48]. Manual gap adjustments were done to improve the alignment and ambiguously aligned regions were also excluded. We finally obtained the combined sequence matrix (Fasta file) generated by BioEdit v.7.0, containing 1777 nucleotide positions from two genes (761 from *rpb*2, 1016 from *tef*1), and the matrix was uploaded to TreeBASE (www.treebase.org (accessed on 5 June 2021).; accession number: S28206).

Maximum likelihood (ML) analysis was computed by using RAxML [49] with the PHY files generated with CLUSTAL_X version 1.83 [47], using the GTR-GAMMA model. Maximum likelihood bootstrap proportions (MLBP) were also computed with 1000 replicates. Bayesian inference (BI) analysis was conducted by using MrBayes 3.1.2 [50] with the NEXUS file converted MEGA6 [51]. The Akaike information criterion (AIC) implemented in jModelTest version 2.0 [52] was used to select the best fit models after likelihood score calculations were done. TPM1uf + I + G was estimated as the best-fit model under the output strategy of AIC. Metropolis-coupled Markov chain Monte Carlo (MCMCMC) searches were run for 5,000,000 generations sampling every 500th generation. Two independent analyses with four chains each (one cold and three heated) were run until stationary distribution was achieved. The initial 25% of the generations of MCMC sampling were excluded as burn-in. The refinement of the phylogenetic tree was used for estimating Bayesian inference posterior probability (BIPP) values. The tree was viewed in TreeView 1.6.6 [53] with maximum likelihood bootstrap proportions (MLBP) greater than 70% and Bayesian inference posterior probabilities (BIPP) greater than 90%, as shown at the nodes.

## 3. Results

### 3.1. Multi-Locus Phylogeny

**Figure 1:**
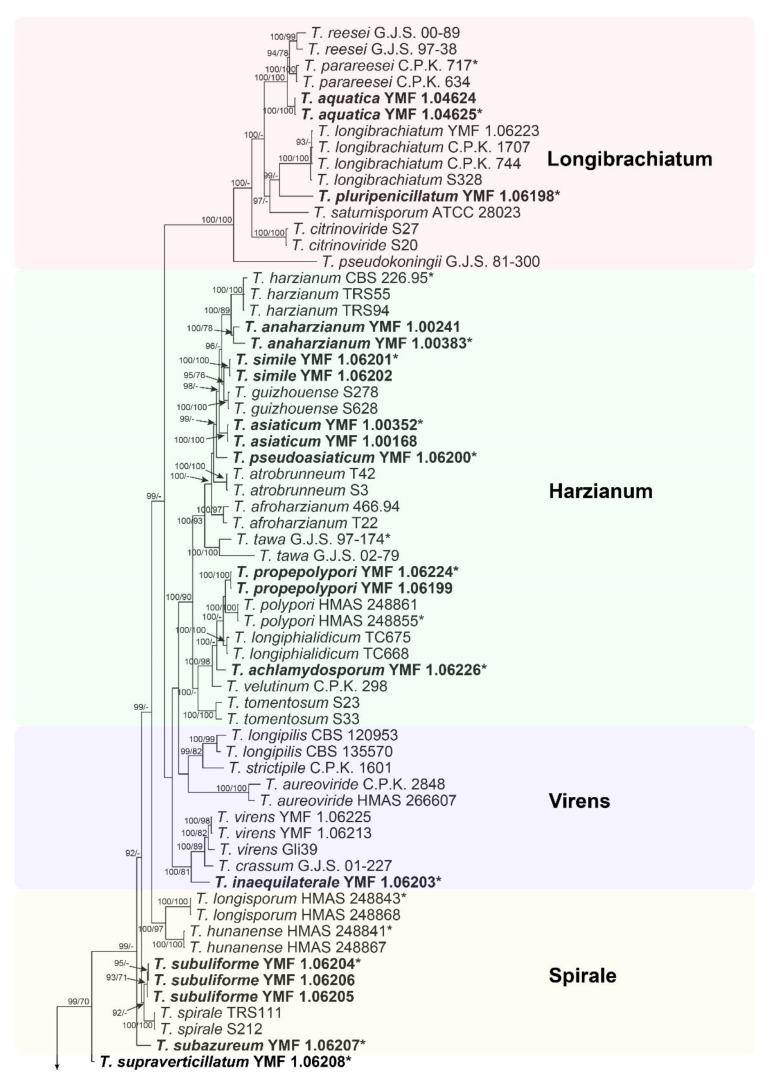

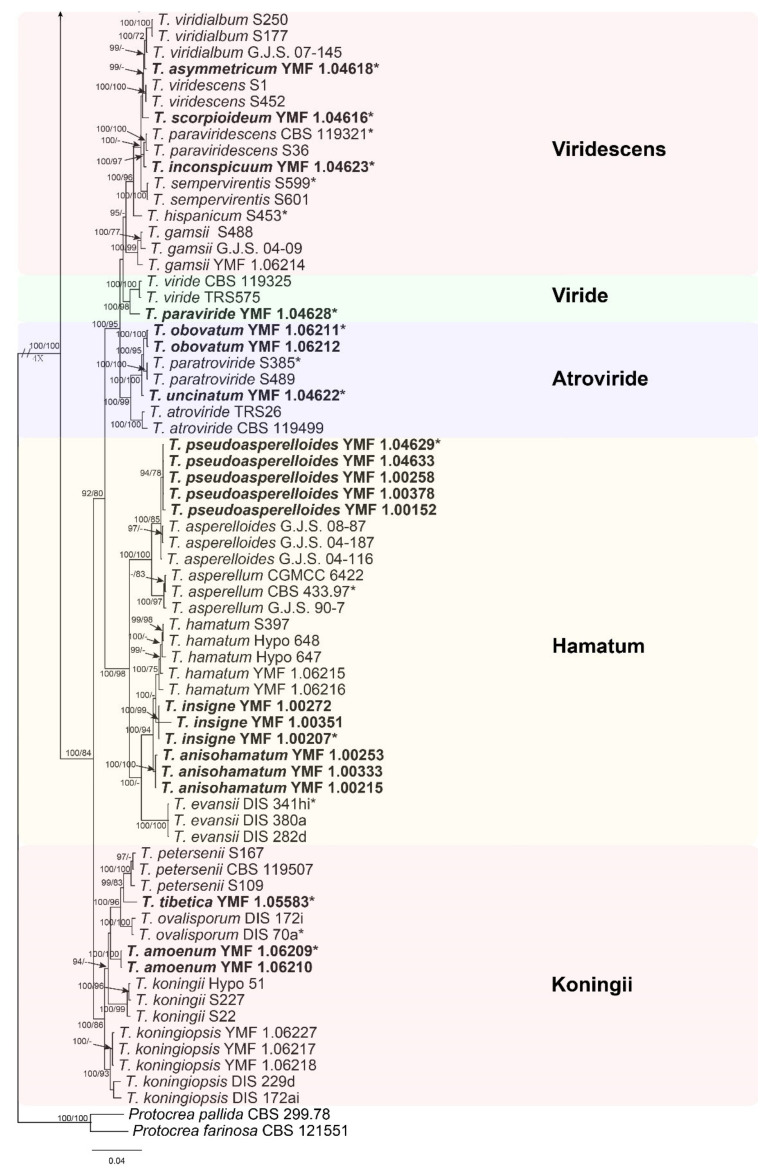
Phylogenic tree generated by the maximum likelihood analysis using combined sequences of *tef*1 and *rpb*2 loci of the genus *Trichoderma*. Bayesian posterior probability values ≥90% (**left**) and Bootstrap values ≥70% (**right**) are indicated at nodes (BIBP/MLBP). *Protocrea farinose* CBS 121551 and *P. pallida* CBS 299.78 were used as the outgroup. Novel species introduced in this study are indicated in bold. The type and ex-type strains are indicated with * after the strain number.

To place these *Trichoderma* isolates, the sequences of *rpb*2 and *tef*1 regions from 83 strains, representing nine clades of *Trichoderma*, were analyzed by the methods of ML and BI, with *Protocrea pallida* CBS 299.78 and *Protocrea farinosa* CBS 121551 as the outgroup. The individual sequence datasets did not show any conflicts in the tree topologies for the 70% reciprocal bootstrap trees, which allowed the two genes for the multi-locus analysis to be combined. The ML analysis showed similar tree topology and was congruent with that obtained in the BI analysis (Figure 1). However, the support values with BI analysis are relatively higher than the ML bootstrap support values. 

In our phylogenetic analysis, the 40 selected isolates (in bold) were distributed among nine independent clades: three from the *Longibrachiatum* clade, ten from the *Harzianum* clade, one from the *Virens* clade, four from the *Spirale* clade, three from the *Viridescens* clade, one from the *Viride* clade, three from the *Atroviride* clade, eleven from the *Hamatum* clade, three from the *Koningii* clade, and one forming its own clade. 

After detailed observations of morphological features, our 40 isolates were considered as 23 new species in *Trichoderma*. Three isolates were located in the *Longibrachiatum* clade, and all species of the *Longibrachiatum* clade formed a monophyletic group with high statistical support (BIBP/MLBP = 100%/100%). The three isolates formed two new subclades corresponding to two new species, designated as *T. aquatica* (BIBP/MLBP = 100%/100%) and *T. pluripenicillatum* (BIBP = 99%). In the *Harzianum* clade, our isolates formed six new subclades corresponding to six new species, designated as *T. anaharzianum* (BIBP/MLBP = 100%/78%), *T. simile* (BIBP/MLBP = 100%/100%), *T. asiaticum* (BIBP/MLBP = 100%/100%), *T. pseudoasiaticum* (BIBP = 99%), *T. propepolypori* (BIBP/MLBP = 100%/100%), and *T. achlamydosporum* (BIBP = 100%). The *Virens* clade contained one new species, designated as *T. inaequilaterale* (BIBP/MLBP = 100%/81%). In the *Spirale* clade, four isolates formed two new subclades corresponding to two new species, designated as *T. subuliforme* (BIBP/MLBP = 93%/71%) and *T. subazureum* (BIBP = 99%). Three isolates in the *Viridescens* formed three new subclades corresponding to three new species, designated as *T. asymmetricum* (BIBP = 99%), *T. inconspicuum* (BIBP/MLBP = 100%/97%), and *T. scorpioideum*. The *Viride* clade contained one new species, designated as *T. paraviride* (BIBP/MLBP = 100%/98%). Three isolates in the *Atroviride* clade formed two new subclades corresponding to two new species, designated as *T. obovatum* (BIBP/MLBP = 100%/100%) and *T. uncinatum* (BIBP/MLBP = 100%/100%). In the *Hamatum* clade, our isolates formed three new subclades corresponding to three new species, designated as *T. pseudoasperelloides* (BIBP/MLBP = 94%/78%), *T. insigne* (BIBP/MLBP = 100%/99%), and *T. anisohamatum* (BIBP/MLBP = 100%/100%). Three isolates in the *Koningii* clade formed two new subclades corresponding to two new species, designated as *T. tibetica* (BIBP/MLBP = 99%/83%) and *T. amoenum* (BIBP/MLBP = 100%/100%). In addition, one isolate was shown as a separate lineage associated with *Spirale, Virens, Harzianum,* and *Longibrachiatum* clades of *Trichoderma*, and further clustered with other species of the genus receiving relatively low supports (BIBP/MLBP = 99%/70%). The isolate was proposed as a new species, designated as *T. supraverticillatum*.

### 3.2. Taxonomy

**Figure 2:**
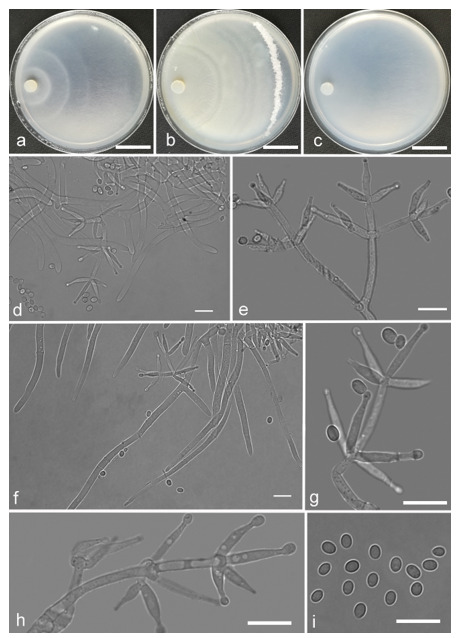
*Trichoderma achlamydosporum* (YMF 1.06226). (**a**–**c**) Cultures after 7 d at 25 °C (**a**) on CMD; (**b**) on PDA; (**c**) on SNA). (**d**–**h**) Conidiophores, phialides and conidia formed on SNA. (**i**) Conidia. Scale bars: (**a**–**c**) = 2.5 cm, (**d**–**i**) = 10 μm.

*Trichoderma achlamydosporum* Z.F. Yu & Y.F. Lv, sp. nov. (Figure 2)

MycoBank MB 834558

Etymology. Latin, *a*-, meaning without, not, -*chlamydosporum*, referred to the chlamydospore.

Type: CHINA, Yunnan province, Jianshui county, N23°15′16″, E102°57′11″, 1170 m alt., from soil, June 2018, Z.F. Yu, Y.F. Lv. Holotype YMF 1.06226, preserved in a metabolically inactive state (deep freezing) in the Conservation and Utilization of Bio-Resources in Yunnan. Ex-type culture CCTCC AF 2021065.

*Conidiophores* straight or slightly curved, comprising an indistinguishable main axis and relatively few distantly placed side branches of varying length, branches sparsely disposed, mostly asymmetrically arranged, also paired, or sometimes at irregular intervals along the central axis, not only once again branched, mostly 1–2 steps, often orientated towards the conidiophore terminus, short. *Phialides* solitary, narrowly lageniform to lanceolate, often asymmetric, straight, less commonly curved or sinuous, generally with long necks, paired or in whorls of 2–4, (6.1–)7.1–13.9(–14.9) × (2.0–)2.4–3.2(–3.5) μm, l/w ratio (2.0–)2.5–6.7 (–7.3), 1.6–2.4(–2.7) μm-wide at the base. *Conidia* oval to ellipsoid, less commonly subglobose or oblong, green, smooth, 3.2–4.2 × 2.3–3.0 μm, l/w ratio 1.2–1.5.

Culture characteristics: Optimum temperature for growth 25 °C. No growth at 35 °C in PDA, CMD, and SNA. Colony radius on CMD after 72 h: 38 mm at 25 °C, 27 mm at 30 °C, covering the plate after 6 days at 25 °C, white zonation conspicuous. Colonies hyaline, circular, mycelium dense, formed reticular texture. Aerial hyphae scant, only found along the colony margin. Chlamydospores were unobserved. No diffusing pigment noted, odor indistinct. Colony radius on PDA after 72 h: 43 mm at 25 °C, 21 mm at 30 °C, covering the plate after 6 days at 25 °C, zonation conspicuous. Colonies translucent, mycelium dense, forming a membraniform covering layer on the medium. Aerial hyphae formed, white, sterile belt that 8 mm from colony margin. Chlamydospores unobserved. Light yellow pigments noted after 4 days. No distinct odor noted. Colony radius on SNA after 72 h: 33 mm at 25 °C, 27 mm at 30 °C, covering the plate after 6 days at 25 °C. Colonies characteristics resemble to those on CMD, but with denser mycelium.

Notes: Phylogenetically, *Trichoderma achlamydosporum* is close to *T. longiphialidicum* Q.V. Montoya et al., and *T. polypori* K. Chen & W.Y. Zhuang. However, the growth rate of *T. achlamydosporum* is slower than *T. longiphialidicum* at 25 °C on PDA, CMD, and SNA. Moreover, *T. achlamydosporum* has shorter phialides [(6.1–)7.1–13.9(–14.9) μm vs. 7.0–21 μm] and larger conidia (3.2–4.2 × 2.3–3.0 μm vs. 2.5–3.5 × 1.6–2.4 μm) than *T. longiphialidicum* [54]. *T. polypori* is different from *T. achlamydosporum* by having more complex branches, longer phialides [(8.9–)11.7–16.6(−20.0) μm], and wider conidia (2.5–3.3 μm) [44]. In addition, *T. longiphialidicum* and *T. polypori* can grow at 35 °C and were observed the production of chlamydospores.

**Figure 3:**
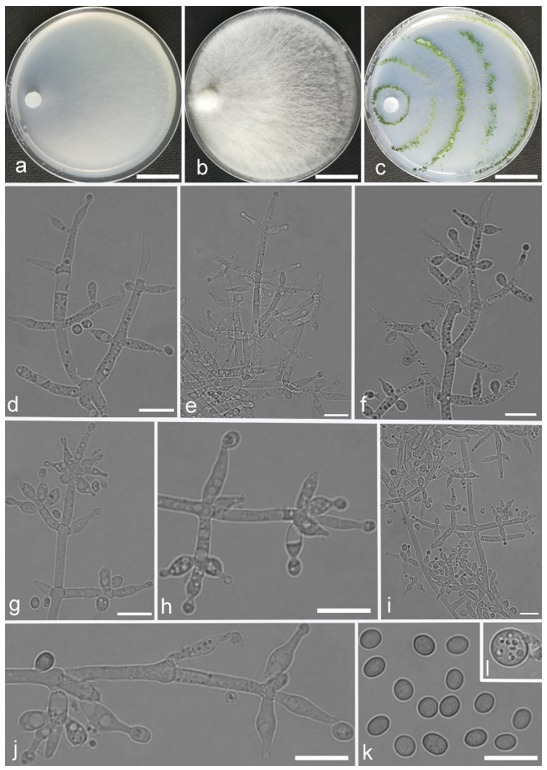
*Trichoderma amoenum* (YMF 1.06209). (**a**–**c**) Cultures after 7 d at 25 °C (**a**) on CMD; (**b**) on PDA; (**c**) on SNA). (**d**–**j**) Conidiophores, phialides and conidia formed on SNA. (**k**) Conidia. Scale bars: (**a**–**c**) = 2.5 cm, (**d**–**k**) = 10 μm.

*Trichoderma amoenum* Z.F. Yu & Y.F. Lv, sp. nov. (Figure 3)

MycoBank MB 834569

Etymology. Latin, amoenum, meaning beautiful, pleasing.

Type: CHINA, Yunnan province, Jianshui county, N23°15′17″, E102°57′15″, 1170 m alt., from soil, June 2018, Z.F. Yu, Y.F. Lv. Holotype YMF 1.06209, preserved in a metabolically inactive state (deep freezing) in the Conservation and Utilization of Bio-Resources in Yunnan. Ex-type culture CCTCC AF 2021068.

*Conidiophores verticillium*-like, straight or slightly curved, paired or unpaired, rebranching on 1–2 levels, sometimes substituted by phialides singly or in whorls of 3–5(–7). *Phialides* ampuliform to tube, few circular with homogeneous necks, widest around the middle, (3.6–)4.3–10.0(–14.9) × (2.1–)2.5–3.8(–4.5) μm, l/w ratio (1.0–)1.4–4.6(–5.8), 1.4–2.9(–3.7) μm wide at the base. *Conidia* smooth, green, oval, subspheroidal, (3.0–)3.7–4.5(–5.0) × 3.2–3.9 μm, l/w ratio 1.0–1.4. *Chlamydospores* globose, smooth, terminal, 6.7–8.9 × 5.5–8.7 μm. 

Culture characteristics: Optimum temperature for growth 25 °C. Colony radius on CMD after 72 h: 55 mm at 25 °C, 47 mm at 30 °C, covering the plate after 5 days at 30 °C, attaining 2 mm after 7 days at 35 °C. Colonies hyaline, mycelia forming subsurface around the inoculum but aerial hyphae present at the margin. Aerial hyphae scanty, and coiling slightly. No diffusing pigment noted, odor indistinct. Colony radius on PDA after 72 h: cover the plate at 25 °C, 55 mm at 30 °C, attaining 13 mm after 7 days at 35 °C. Colonies white, radial, loose. Aerial hyphae dense, clustered on the outer layer of the colony and extending to the Petri dish lid that gives the colony a downy texture. No diffusing pigment noted, odor indistinct. Colony radius on SNA after 72 h: 42 mm at 25 °C, 22 mm at 30 °C, covering the plate after 5 days at 25 °C. Colonies green, circular, radial, zonate. Aerial hyphae scant, inconspicuous. Conidiation formed in pustules after 2 days, pustules abundant in kelly and bottle-green bands, spreading in 4–5 concentric rings. No diffusing pigment noted, odor indistinct. 

Additional specimen examined: CHINA, Yunnan province, Jianshui county, N23°15′17″, E102°57′15″, 1170 m alt., from soil, June 2018, Z.F. Yu, Y.F. Lv., living culture YMF 1.06210.

Notes: Phylogenetically, *Trichoderma amoenum* is closely related to *T. koningii* Oudemans & Koning and *T. koningiopsis.* However, *T. amoenum* can be distinguished from them in morphology. *T. amoenum* forms intricately branched conidiophores with branches that bear numerous ampuliform phialides in whorls of 3–5(–7) and has smooth, oval to subspheroidal conidia, while *T. koningii* has lageniform phialides in whorls of 3–4 and oblong conidia [55]. *T. koningiopsis* resembles *T. amoenum* by having similar conidiophores, but has narrower conidia (2.2–3.0 μm vs. 3.2–3.9 μm) [55].

**Figure 4:**
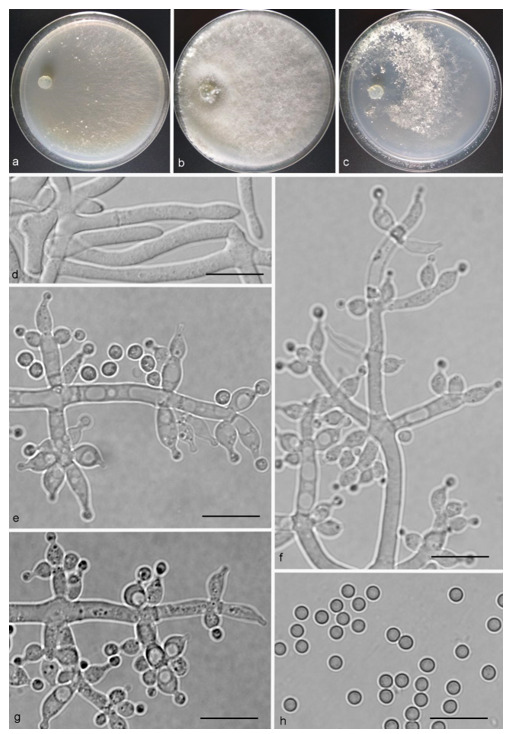
*Trichoderma anaharzianum* (YMF 1.00383). (**a**–**c**) Cultures after 7 d at 25 °C (**a**) on CMD; (**b**) on PDA; (**c**) on SNA). (**d**) Conidiophores formed on SNA. (**e**–**g**) Conidiophores, phialides and conidia formed on SNA. (**h**) Conidia. Scale bars: (**a**–**c**) = 2.5 cm, (**d**–**h**) = 10 μm.

*Trichoderma anaharzianum* Z.F. Yu & X. Du, sp. nov. (Figure 4)

MycoBank MB825471

*Etymology*. Greek, *ana*-, meaning up to, toward, exceedingly, back, against, -*harzianum*, referring to the species *Trichoderma harzianum*.

Type: CHINA, Yunnan province, Luliang county, N24°57′22″, E103°46′30″, 1800 m alt., from soil of tobacco rhizosphere, July 2007, Z.F. Yu, Y.F. Lv. Holotype YMF 1.00383, preserved in a metabolically inactive state (deep freezing) in the Conservation and Utilization of Bio-Resources in Yunnan. Ex-type culture CGMCC 3.19086.

*Conidiophores* straight or slightly curved, numerous, branched, commonly contain paired branches, re-branching 1–2 times, 3.0–5.0 μm wide at base, the distance between two neighboring branches is (15.0–)19.0–23.0(–27.0) μm. The branches generally perpendicular to main axis, terminating in 3–7 phialides. *Phialides* ampulliform to lageniform in a verticillate fashion, sometimes arising singly directly from the main axis, (4.0–)5.0–6.0(–7.0) × 2.0–4.0 μm, l/w ratio (1.0–)1.3–2.0(–2.3). *Conidia* globose to subglobose, thin-walled, green, smooth, (2.5–)2.6–3.1(–3.2) × (2.1–)2.2–2.8(–3.0) μm, l/w ratio (1.0–)1.1–1.3(–1.4).

Culture characteristics: Optimum temperature for growth 25 °C. Colony radius on CMD after 72 h: cover the plate at 25 °C, 38 mm at 30 °C, attaining 33 mm after 7 days at 35 °C. Colonies homogenous, translucent, radial. Aerial hyphae distinct, loosely aggregated. Conidiation formed on minute pustules after 3 days, pustule surface with roundish or irregular outline, successively turning green after 5 days. Chlamydospores unobserved. No diffusing pigment noted, odor indistinct. Colony radius on PDA after 72 h: 64–76 mm at 25 °C, 50 mm at 30 °C, 11–18 mm at 35 °C, covering the plate after 4 days at 25 °C. Colonies circular and dense. Aerial hyphae abundant, whitish arachnoid, Conidia noted after 3 days, and successively turning green after 4 days. Chlamydospores unobserved. No diffusing pigment noted, odor indistinct. Colony radius on SNA after 72 h: 46–53 mm at 25 °C, 30–35 mm at 30 °C, 6 mm at 35 °C, covering the plate after 6 days at 25 °C. Colonies hyaline, loose, radial, indistinctly zonate. Aerial hyphae white and sparse, mostly around the center of colony, conidia noted after 4 days, gradually turning green after 5 days. Chlamydospores unobserved. No diffusing pigment noted, odor indistinct. 

Additional specimen examined: CHINA, Yunnan province, Luliang county, N24°57′22″, E103°46′30″, 1800 m alt., from soil of tobacco rhizosphere, July 2007, Z.F. Yu, living culture YMF 1.00241.

Notes: *Trichoderma anaharzianum* is phylogenetically closely related to *T. harzianum* Rifai. Morphologically, *T. anaharzianum* has a wider branch base. Moreover, the conidia of *T. harzianum* forms from thick and dense concentric ring on PDA and SNA, or uniformly throughout the colony [56], while this was not observed on *T. anaharzianum*.

**Figure 5:**
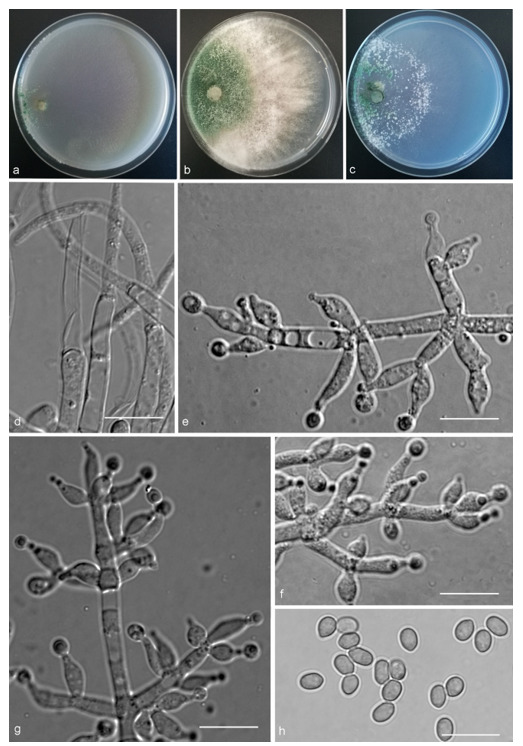
*Trichoderma anisohamatum* (YMF 1.00333). **(a**–**c**) Cultures after 7 d at 25 °C (**a**) on CMD; (**b**) on PDA; (**c**) on SNA). (**d**) Conidiophores formed on SNA. (**e**–**g**) Conidiophores, phialides and conidia formed on SNA. (**h**) Conidia. Scale bars: (**a**–**c**) = 2.5 cm, (**d**–**h**) = 10 μm.

*Trichoderma anisohamatum* Z. F. Yu & X. Du, sp. nov. (Figure 5)

MycoBank MB825467

*Etymology*. Greek, *aniso*-, meaning unequal, uneven, -*hamatum*, referring to the species *Trichoderma hamatum*.

Type: CHINA, Yunnan province, Luliang county, N24°57′22″, E103°46′30″, 1800 m alt., from soil of tobacco rhizosphere, July 2007, Z.F. Yu. Holotype YMF 1.00333, preserved in a metabolically inactive state (deep freezing) in the Conservation and Utilization of Bio-Resources in Yunnan. Ex-type culture CGMCC 3.19083.

*Conidiophores* regularly dendriform, straight or slightly curved, comprising a distinct main axis with side branches paired or unilateral and often terminating in whorl of 3–5 divergent phialides, rarely in whorl of 7, 3.0–4.0(–5.0) μm wide at the base, the distance between two neighbor branches is (19.0–)20.0–24.0(–26.0) μm. *Phialides* mostly lageniforms, spindly, sometimes with cylindrical bent neck, fasciculate near the tip of main axis, occasionally solitary, 5.0–8.0 × 3.0–4.0(–5.0) μm, l/w ratio (1.0–)1.3–2.3(–2.7). *Conidia* subglobose to globose, sometimes ellipsoidal, green, smooth, (3.6–)3.8–4.6(–4.8) × (2.7–)2.8–3.3(–3.4) μm, l/w ratio (1.1–)1.2–1.6(–1.7).

Culture characteristics: Optimum temperature for growth 25 °C. No growth at 35 °C in PDA, CMD and SNA. Colony radius on CMD after 72 h: 51–59 mm at 25 °C, 16 mm at 30 °C, covering the plate after 4 days at 25 °C. Colonies hyaline, circular, margin distinct. Aerial hyphae inconspicuous. Conidiation formed in pustules after 3 days, pustules relatively sparse, spreading on the side of inoculum. Chlamydospores unobserved. No diffusing pigment noted, odor indistinct. Colony radius on PDA after 72 h: 64–68 mm at 25 °C, 16 mm at 30 °C, covering the plate after 4 days at 25 °C. Colonies layered distinctly, green in the center, white at the margin, radial. Aerial hyphae hairy to floccose, dense on inner layer, but relative sparse on the margin, a large green disk around the inoculums. Chlamydospores unobserved. No diffusing pigment noted, odor indistinct. Colony radius on SNA after 72 h: 45–49 mm at 25 °C, 10 mm at 30 °C, covering the plate after 5 days at 25 °C. Colonies green in center, white at the margin, margin well-fined. Aerial hyphae sparse on outer layer, radiated indistinctly. Conidiation formed in pustules after 3 days, pustules abundant, originally white and gradually turning green. Chlamydospores unobserved. No diffusing pigment noted, odor indistinct.

Additional specimens examined: China, Yunnan province, Yunnan province, Luliang county, N24°57′22″, E103°46′30″, 1800 m alt., from soil of tobacco rhizosphere, July 2007, Z.F. Yu, living culture YMF 1.00253, YMF 1.00333.

Notes: *Trichoderma anisohamatum* differs morphologically from the phylogenetically nearest species *T. hamatum* by its sterile secondary branches. Furthermore, the phialides of *T. hamatum* are somewhat swollen in the middle [57].

**Figure 6:**
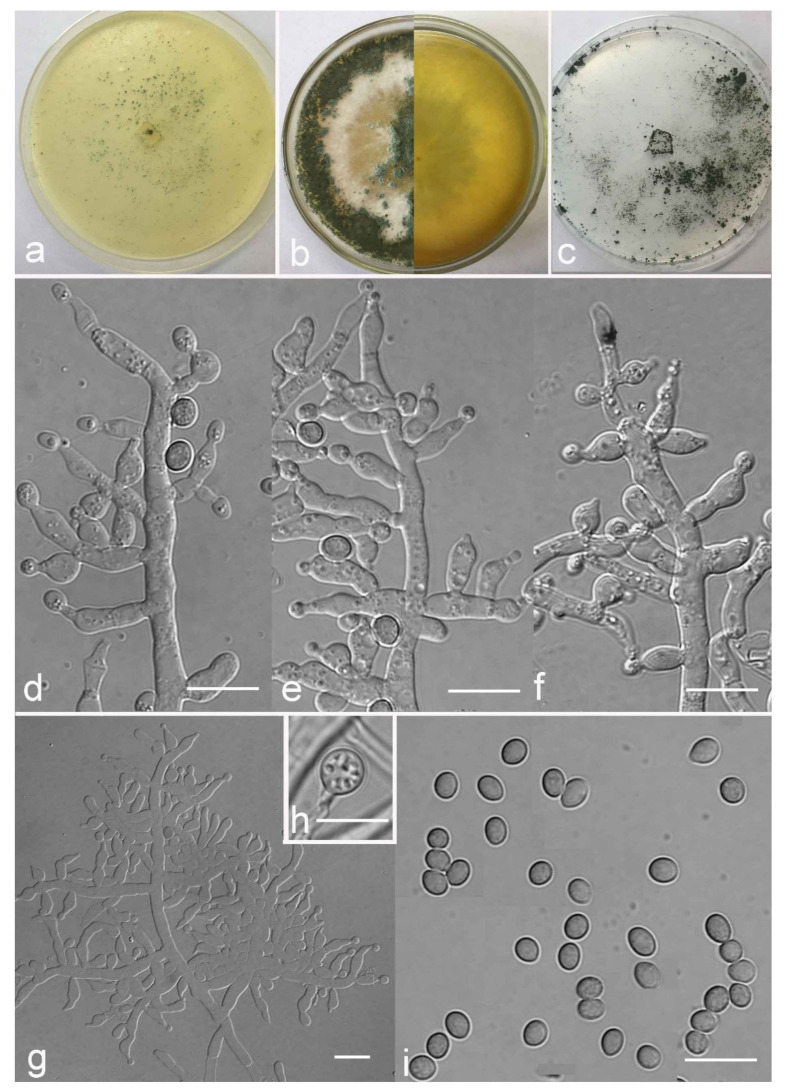
*Trichoderma aquatica* (YMF 1.04625). (**a**–**c**) Cultures after 7 d at 25 °C (**a**) on CMD; (**b**) on PDA; (**c**) on SNA). (**d**–**g**) Conidiophores, phialides and conidia formed on SNA. (**h**) Chlamydospores. (**i**) Conidia. Scale bars: (**a**–**c**) = 2.5 cm, (**d**–**i**) = 10 μm.

*Trichoderma aquatica* Z.F. Yu & X. Du, sp. nov. (Figure 6)

MycoBank MB830633

*Etymology.* Latin, *aquatica*, referring to the isolated source from water environment.

Type: CHINA, Sichuan province, Luhuo county, N31°39′7.5″, E100°15′31.9″, 3507 m alt., endophytic in living root of *Batrachium bungei*, July 2014, Z.F. Yu, Y. Huang. Holotype YMF 1.04625, preserved in a metabolically inactive state (deep freezing) in the Conservation and Utilization of Bio-Resources in Yunnan. Ex-type culture CGMCC 3.19077.

*Conidiophores* macronematous, mononematous, pyramidal aspect, alternately or irregular branched, often terminating in a verticil of the 3–5 phialides, the distance between two neighboring branches is 5.5–19.1 μm. *Phialides* lageniform, rare ampulliform, slightly curved, 5.2–11.5 × (2.5–)3.1–4.2 μm, l/w ratio 1.1–2.8(–3.0), discrete, perpendicular to the main axis or integrated terminal arranged, sometimes arising in crowded and compact fascicles or singly directly from the main axis, 2.4–4.4 μm wide at base. *Conidia* mostly ovoid, ellipsoidal, rarely subglobose, thin-walled, green, smooth, 3.3–4.8 × 2.4–3.3 μm, l/w ratio 1.1–1.6. *Chlamydospores* globose, smooth, terminal, 4.3–7.6 × 3.91–7.2 μm.

Culture characteristics: Colony grows very fast on CMD and PDA. Colony radius on CMD after 72 h: cover the plate at 25 °C, 30 °C, and 35 °C. Colonies hyaline, thin, mycelium sparse. Aerial hyphae sparse, scant, inconspicuous. Conidiation minute, formed in pustules after 3 days, pustules relatively sparse, spreading uniformly throughout the colony, turning dark green after 4 days. Light yellow pigments noted after 3 days. No distinct odor noted. Colony radius on PDA after 72 h: cover the plate at 25 °C, 30 °C, and 35 °C. Colonies white in the center, dark green in margin, circular, zonate distinctly. Arial hyphae radial distinctly, abundant, dense, gradually forms a continuous lawn, granulated to pulvinate pustules on the margin and central around the inoculums. Conidiation formed in pustules after 3 days, pustules abundant, gradually turning dark green after 4 days. Yellow pigments noted distinctly. No distinct odor noted. Colony radius on SNA after 72 h: 49–55 mm at 25 °C, 47–60 mm at 30 °C, 34–40 mm at 35 °C, covering the plate after 5 days at 25 °C. Colonies not zonate, hyaline. Aerial hyphae sparse, scant, inconspicuous and radial indistinctly. Conidiation formed in pustules after 2 days, pustules minute, compact, scattered, turning dark green rapidly. No diffusing pigment noted, odor indistinct.

Additional specimen examined: CHINA, Sichuan province, Luhuo county, N31°39′7.5″, E100°15′31.9″, 3507 m alt., endophytic in living stem of *Batrachium bungei*, July 2014, Z.F. Yu, Y. Huang, living culture YMF 1.04624.

Notes: The phylogenetic analyses based on *rpb2* and *tef1* reveal that *Trichoderma aquatica* is related to *T. parareesei* Jaklitsch et al. and *T. reesei* E.G. Simmons. *T. aquatica* is morphologically most similar to *T. parareesei* in the loosely arranged and unpaired branches, cylindrical-neck phialides and smooth, green conidia, whereas *T. parareesei* is obviously distinguished by lageniform, shorter and narrower phialide [(4.5–)5.0–8.0(–11.0) × (2.5–)2.7–3.5(–3.8) μm], and uniformly ellipsoidal, longer and wider conidia [(3.3–)3.8–4.5(–6.2) × (2.5–)2.8–3.2(–3.5) μm] [58]. Furthermore, *T. reesei* has narrower phialides (2.2–)2.5–3.5(–4.0) μm and longer conidia 3.5–6.0 (–9.0) μm [58], which distinctly differs from mostly ovoid conidia of the new species.

**Figure 7:**
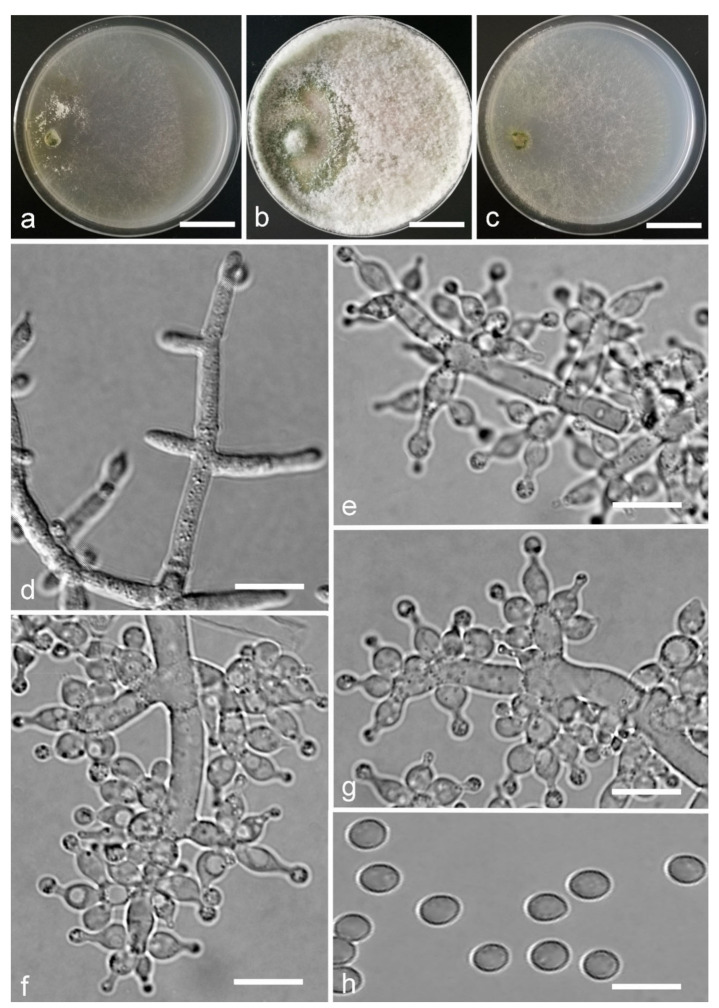
*Trichoderma asiaticum* (YMF 1.00352). **(a**–**c**) Cultures after 7 d at 25 °C (**a**) on CMD; (**b**) on PDA; (**c**) on SNA). (**d**) Conidiophores formed on SNA. (**e**–**g**) Conidiophores, phialides and conidia formed on SNA. (**h**) Conidia. Scale bars: (**a**–**c**) = 2.5 cm, (**d**–**h**) = 10 μm.

*Trichoderma asiaticum* Z.F. Yu & X. Du, sp. nov. (Figure 7)

MycoBank MB825470

*Etymology*. Latin, *asiaticum*, referring to the continent of Asia.

Type: CHINA, Yunnan province, Luliang county, N24°57′22″, E103°46′30″, 1800 m alt., from soil of tobacco rhizosphere, July 2007, Z.F. Yu, Y.F. Lv. Holotype YMF 1.00352, preserved in a metabolically inactive state (deep freezing) in the Conservation and Utilization of Bio-Resources in Yunnan. Ex-type culture CGMCC 3.19085.

*Conidiophores* typically comprise a distinct main axis with one terminal whorl of 4–5 phialides and mostly paired side branches. Branches mostly are perpendicular to the main axis, with septa inconspicuous. The distance between two neighboring branches is (5.0–)6.0–22.0(–24.0) μm. Base not well-defined, about 2.0–4.0 μm wide. Each branch terminating in a whorl of up to 3–5 phialides. *Phialides* ampulliform to lageniform, (3.0–)4.0–6.0(–7.0) × (1.0–)2.0–3.0(–4.0) μm, l/w ratio (1.0–)1.3–3.0(–4.0), usually verticillated around branch. *Conidia* commonly subglobose to globose, oblong rarely noted, green, smooth, (2.3–)2.4–3.0(–3.1) × (2.0–)2.1–2.7(–2.8) μm, l/w ratio (1.0–)1.1–1.3(–1.4).

Culture characteristics: Optimum temperature for growth 25 °C. Colony radius on CMD after 72 h: 55 mm at 25 °C, 30–35 mm at 30 °C, 9–13 mm at 35 °C, covering the plate after 4 days at 25 °C. Colonies hyaline, outline distinct, fan-shaped. Aerial hyphae loose, sparse, radial and arachnoid, branched distinctly. Pustules white, minute, distributed in a scattered fashion around the point of inoculation, turning green after 5 days. Chlamydospores unobserved. Light yellow pigments noted. No distinct odor noted. Colony radius on PDA after 72 h: 54 mm at 30 °C, 12 mm at 35 °C, covering the plate after 3 days at 25 °C. Colonies thick and dense, the zone around the central part of colony forms a distinct circular and green part. Aerial hyphae whitish, partly green, abundant, floccose to cottony, zonate distinctly. Chlamydospores unobserved. No diffusing pigment noted, odor indistinct. Colony radius on SNA after 72 h: 58–65 mm at 25 °C, 37 mm at 30 °C, 7 mm at 35 °C, covering the plate after 4 days at 25 °C. Colonies similar to that on CMD, regular and distinct outline, but translucent and round-like. Aerial hyphae branched, loose and sparse, radial and arachnoid. Pustules white, minute, scattered around the point of inoculation, gradually turning green after 3 days. Chlamydospores unobserved. Light yellow pigments noted. No distinct odor noted.

Additional specimen examined: CHINA, Yunnan province, Luliang county, N24°57′22″, E103°46′30″, 1800 m alt., from soil of tobacco rhizosphere, July 2007, Z.F. Yu, living culture YMF 1.00168.

Notes: *Trichoderma asiaticum* is a member of the *Harzianum* clade. Phylogenetically, *T. asiaticum* is closely related to *T. afroharzianum* Chaverri & Jaklitsch and *T. atrobrunneum* Chaverri & Jaklitsch, but the branch base of the two species are narrower [(1.0–)1.5–2.2(–3.5) and 1.0–2.7 mm, respectively][56]. In addition, some phialides of *T. asiaticum* are somewhat shorter than those of *T. afroharzianum* [(3.0–)4.0–6.0(–7.0) vs. (3.5–)5.2–10.2(–17.5) mm]. For the *T. atrobrunneum*, the base of the branch is narrower than that of *T. asiaticum* and the conidia of the *T. atrobrunneum* are subglobose to ovoid [56], which significantly differs from the globose and smaller conidia of *T. asiaticum* [(2.3–)2.4–3.0(–3.1) × (2.0–)2.1–2.7(–2.8) μm].

**Figure 8:**
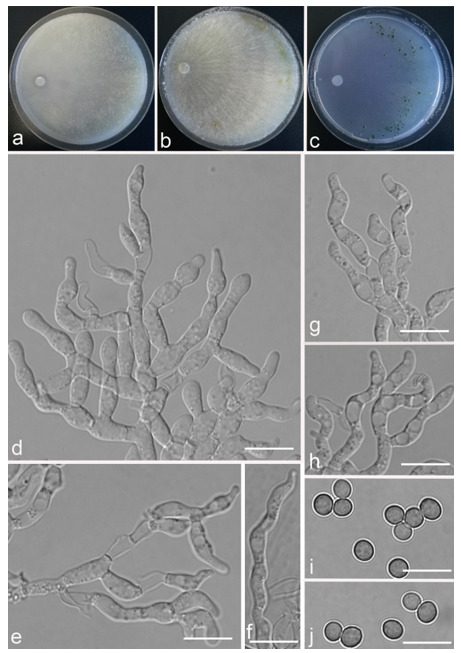
*Trichoderma asymmetricum* (YMF 1.04618). (**a**–**c**) Cultures after 7 d at 25 °C (**a**) on CMD; (**b**) on PDA; (**c**) on SNA). (**d**–**h**) Conidiophores and phialides formed on SNA. (**i**–**j**) Conidia. Scale bars: (**a**–**c**) = 2.5 cm, (**d**–**j**) = 10 μm.

*Trichoderma asymmetricum* Z.F. Yu & X. Du, sp. nov. (Figure 8)

MycoBank MB830637

*Etymology*. Latin, *asymmetricus,* referring to the asymmetrically arranged branches.

Type: CHINA, Sichuan province, Daocheng county, N29°29′48.5″, E100°14′46.5″, 4362 m alt., endophytic in living leaf of *Hippuris vulgaris* in pond, July 2014, Z.F. Yu, Y. Huang. Holotype YMF 1.04618, preserved in a metabolically inactive state (deep freezing) in the Conservation and Utilization of Bio-Resources in Yunnan. Ex-type culture CGMCC 3.19164.

*Conidiophores* typically tree-like, rarely comprising a main axis with a terminal solitary phialide, often crowded, typically asymmetrically branched. The bases of branches are not well-defined, about 2.3–4.2 μm wide. Branches commonly sinuous and swollen below the phialides, diverging to form 1–3 phialides at the terminal. *Phialides* usually sterile, occasionally lageniform, with the apex generally obtuse, sometimes arising alone from the main axis often with a long base, (4.4–)5.9–11.8(–12.6) × 2.3–4.0(–4.4) μm, l/w ratio (1.3–)1.7–4.3(–5.0). *Conidia* green, smooth, subglobose to globose, sometimes broadly ellipsoidal, rarely ovoid, 3.5–4.5(–5.0) × 3.2–4.1 μm, l/w ratio 1.0–1.4.

Culture characteristics: Optimum temperature for growth 25 °C. No growth at 30 °C in PDA and CMD, and at 35 °C in SNA. Colony radius on CMD after 72 h: 41 mm at 25 °C. Colonies translucent, thin, indistinctly radial around the point of inoculation, conspicuously arachnoid and relatively dense near the margin. Aerial hyphae common, denser in distant areas. Conidiation formed in pustules after 15 days; pustules white, turning green after 23 days. Chlamydospores unobserved. No diffusing pigment noted, odor indistinct. Colony radius on PDA after 72 h: 57 mm at 25 °C, covering the plate after 5 days at 25 °C. Colonies white, circular in outline, compact, indistinctly zonate, with well-defined margin. Aerial hyphae radial conspicuously, dense and extending toward the distal margin. Chlamydospores unobserved. No diffusing pigment noted, odor indistinct. Colony radius on SNA after 72 h: 37–40 mm at 25 °C, less than 10 mm at 30 °C, covering the plate after 8 days at 25 °C. Colonies hyaline, thin, circular, margin obscure. Aerial hyphae sparse, arachnoid, somewhat branched, becoming fertile latter. Pustules arranged regularly, generally in a broad marginal zone, asymmetrical to hemispherical, formed after 13 days and turned green after 14 days. Chlamydospores unobserved. No diffusing pigment noted, odor indistinct.

Notes: Phylogenetic analyses showed that *Trichoderma asymmetricum* is closely related to *T. viridescens* Jaklitsch & Samuels, they both share undiscernible conidiophores and some branches that only have a few phialides. The middle part of phialides of the two species are usually swollen and the necks are long cylindrical. Moreover, the conidia of two species in SNA are mostly subglobose, some ovoid. The biggest difference between two species are that the top of phialides of *T. asymmetricum* is often curved rather than constricted, and mostly sterile, which differs from the narrow phialides top of *T. viridescens*. The phialide of *T. viridescens* generally looks submoniliform. Furthermore, the conidia of *T. asymmetricum* are commonly slightly wider than those of *T. viridescens* [(2.2–)3.0–3.7(–4.7) μm] [59]. *T. asymmetricum* is also phylogenetically close to *T. viridialbum* Jaklitsch et al., and the two species have optimal growth at 25 °C on all media, with restricted growth at 30 °C and no growth at 35 °C. However, *T. viridialbum* has longer phialides (6.8–)8.8–12.7(–15.0) × (2.2–)2.5–3.0(–3.5) μm than *T. asymmetricum* [60].

**Figure 9:**
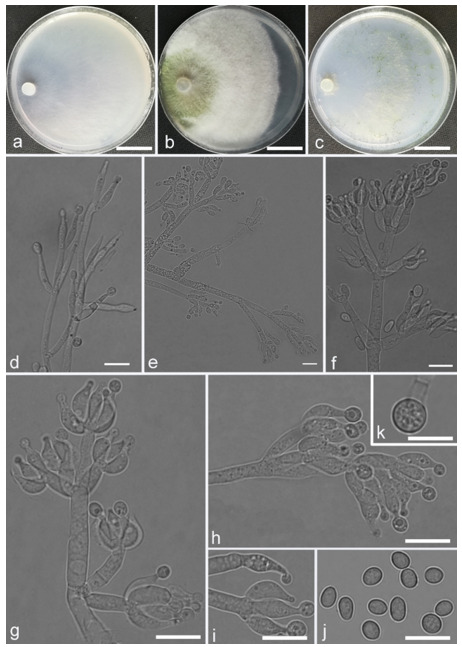
*Trichoderma inaequilaterale* (YMF 1.06203). (**a**–**c**) Cultures after 7 d at 25 °C (**a**) on CMD; (**b**) on PDA; (**c**) on SNA). (**d**–**i**) Conidiophores, phialides and conidia formed on SNA. (**j**) Conidia. (**k**) Chlamydospores. Scale bars: (**a**–**c**) = 2.5 cm, (**d**–**k**) = 10 μm.

*Trichoderma inaequilaterale* Z.F. Yu & Y.F. Lv, sp. nov. (Figure 9)

MycoBank MB 834562

*Etymology*. Latin, *inaequilaterale*, referring to the asymmetric phialides.

Type: CHINA, Yunnan province, Jianshui county, N23°16′22″, E102°57′37″, 1170 m alt., from soil, June 2018, Z.F. Yu, Y.F. Lv. Holotype YMF 1.06203, preserved in a metabolically inactive state (deep freezing) in the Conservation and Utilization of Bio-Resources in Yunnan. Ex-type culture CCTCC AF 2021066.

*Conidiophores penicillium*-like, mostly asymmetrically arranged, below terminal branches also paired or in whorls of 2–3, not only primary branches, mostly 1–3 times at irregular intervals along the central axis, straight or slightly bent, orientating towards the conidiophore terminus. *Phialides* ampuliform or narrowly lageniform, inequilateral, curved, less commonly straight, with long or bent necks in whorls of (1–)2–5, occasionally solitary or paired along conidiophores, asymmetrical, only symmetrical at the conidiophore terminus, (4.2–)6.0–13.6(–19.7) × (2.2–)2.7–4.1(–4.7) μm, l/w ratio (1.4–)1.6–4.3(–5.9), (1.3–)1.6–2.7 μm wide at base, widest around the middle. *Conidia* ellipsoidal to oval, sometimes oblong, light green, smooth, 4.2–5.8 × (3.2–)3.5–4.2(–4.4) μm, l/w ratio 1.1–1.6. *Chlamydospores* generally globose to subglobose, smooth, terminal, 5.6–9.3 × 5.5–9.1 μm. 

Culture characteristics: Colony radius on CMD after 72 h: 62 mm at 25 °C, 58 mm at 30 °C, 10 mm at 35 °C, covering the plate after 4 days at 25 °C. Colonies hyaline, mycelium extend from the inoculation plug, forming a reticulum. Aerial hyphae scant, inconspicuous. No diffusing pigment noted, odor indistinct. Colony radius on PDA after 72 h: 44 mm at 25 °C, 46 mm at 30 °C, 15 mm at 35 °C, covering the plate after 6 days at 25 °C. Colonies white, but greenish yellow around the inoculation plug, zonate, dense. Aerial hyphae abundant, hairy to floccose, a large green disk around the inoculums. No diffusing pigment noted, odor indistinct. Colony radius on SNA after 72 h: 63 mm at 25 °C, 16 mm at 35 °C, covering the plate after 3 days at 30 °C. Colonies hyaline, loose, radial, indistinctly zonate. Aerial hyphae white, abundant on the colony middle. Pustules minute, dispersedly distributed, firstly white, gradually turning green after 3 days. No diffusing pigment noted, odor indistinct.

Notes: Phylogenetic analyses showed that *Trichoderma inaequilaterale* is the closely related species to *T. crassum* Bissett and *T. virens* J.H. Miller et al. Morphologically, *T. inaequilaterale* can be distinguished from *T. crassum* by the size of the phialides (4.2–)6.0–13.6(–19.7) × (2.2–)2.7–4.1(–4.7) μm, whereas the phialides of *T. crassum* are (8.5–)13.5–15.7(–28.0) × (3.3–)4.3–4.6(–5.7) μm [61]. Moreover, *T crassum* is unable to grow at 35 °C. *T. inaequilaterale* grows faster on SNA than on PDA, the same as *T. virens*, but *T. virens* produces yellow-diffusing pigments on CMD and PDA while *T. inaequilaterale* does not [62].

**Figure 10:**
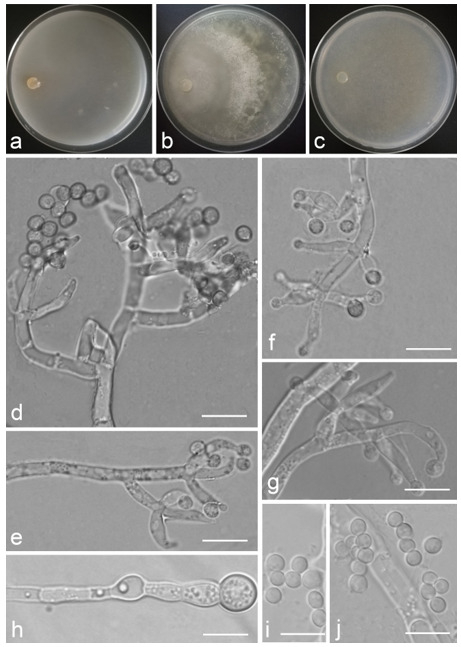
*Trichoderma inconspicuum* (YMF 1.4263). (**a**–**c**) Cultures after 7 d at 25 °C (**a**) on CMD; (**b**) on PDA; (**c**) on SNA). (**d**–**g**) Conidiophores, phialides and conidia formed on SNA. (**h**) Chlamydospores. (**i**–**j**) Conidia. Scale bars: (**a**–**c**) = 2.5 cm, (**d**–**j**) = 10 μm.

*Trichoderma inconspicuum* Z.F. Yu & X. Du, sp. nov. (Figure 10)

MycoBank MB830639

*Etymology*. Latin, *inconspicuum*, meaning not remarkable, not striking, referring to the conidiophores and pialides growing in pure cultures.

Type: CHINA, Yunnan province, Jianchuan county, Jian Lake, N26°28′5.16″, E99°55′4.88″, 2182 m alt., endophytic in living stem of *Nymphoides peltatum*, July 2014, Z.F. Yu, Y. Huang. Holotype YMF 1.04623, preserved in a metabolically inactive state (deep freezing) in the Conservation and Utilization of Bio-Resources in Yunnan. Ex-type culture CGMCC 3.19159.

*Conidiophores* difficult to distinguish between mycelium, tree-like or irregular branches, the branches usually terminating in a whorl of 3–4 phialides, 2.7–4.3 μm wide at the base of branches. *Phialides* commonly narrow lageniform, some swollen in the middle, ampulliform, the neck usually curved, 6.4–14.4(–16.2) × 1.9–4.0 μm, l/w ratio 1.9–6.5. *Conidia* smooth, thin-walled, mostly subglobose to globose, a few ovoid, pale yellow-green, smooth, 2.8–4.5 × 2.6–4.0 μm, l/w ratio 1.0–1.4. *Chlamydospore* common, globose, ellipsoidal, smooth, terminal and intercalary, 6.6–10.1 × 5.8–8.7 μm.

Culture characteristics: Optimum temperature for growth 25 °C. No growth at 30 °C in CMD, PDA and SNA. Colony radius on CMD after 72 h: 27–32 mm at 25 °C, covering the plate after 6 days at 25 °C. Colonies hyaline, margin not well-defined. Aerial hyphae nearly lacking. No diffusing pigment noted, odor indistinct. Colony radius on PDA after 72 h: 48–53 mm at 25 °C, covering the plate after 6 days at 25 °C. Colonies white, flat, zonate indistinctly. Aerial hyphae powdery, hard to picking, sparse on outer layer, relatively compact on inner layer. No diffusing pigment noted, odor indistinct. Colony radius on SNA after 72 h: 29 mm at 25 °C, covering the plate after 7 days at 25 °C. Colonies similar to that on CMD. No diffusing pigment noted, odor indistinct.

Notes: *Trichoderma inconspicuum* is phylogenetically close to *T. paraviridescens* Jaklitsch et al., but their differences in morphology are distinct. The conidiophores of *T. paraviridescens* are typically dendriform, with unpaired and right angle arranged branches, and the phialides is straight lageniform or sometimes curved. However, the phialides of *T. inconspicuum* are hard to distinguish when grow, they are sparsely distributed, usually longer than *T. paraviridescens* (7.3–10.3 μm), and the conidia of *T. paraviridescens* [(3.3–)3.7–4.3(–4.7) μm] are longer than those of *T. inconspicuum* [60]. Morphologically, *T. inconspicuum* is somewhat similar to *T. viridialbum* in having globose to subglobose conidia, but no pustules were observed in *T. inconspicuum* [60].

**Figure 11:**
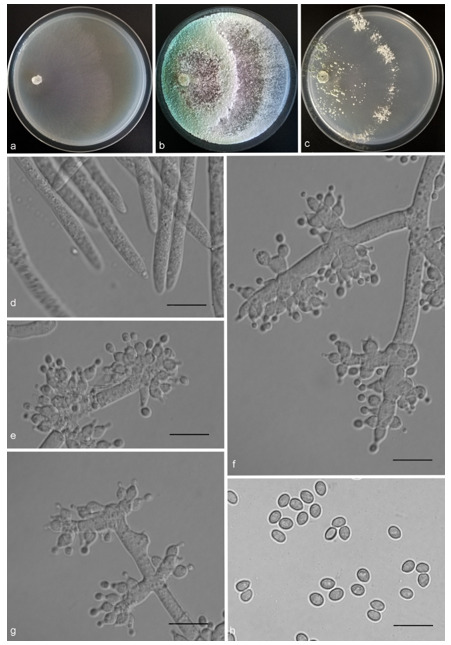
*Trichoderma insigne* (YMF 1.00207). (**a**–**c**) Cultures after 7 d at 25 °C (**a**) on CMD; (**b**) on PDA; (**c**) on SNA). (**d**) Conidiophores formed on SNA. (**e**–**g**) Conidiophores, phialides and conidia formed on SNA. (**h**) Conidia. Scale bars: (**a**–**c**) = 2.5 cm, (**d**–**h**) = 10 μm.

*Trichoderma insigne* Z.F. Yu & X. Du, sp. nov. (Figure 11)

MycoBank MB825464

*Etymology*. Latin, *insigne*, meaning distinguished, remarkable.

Type: CHINA, Yunnan province, Luliang county, N24°57′22″, E103°46′30″, 1800 m alt., from soil of tobacco rhizosphere, July 2007, Z.F. Yu. Holotype YMF 1. 00207, preserved in a metabolically inactive state (deep freezing) in the Conservation and Utilization of Bio-Resources in Yunnan. Ex-type culture CGMCC 3.19080.

*Conidiophores* pyramidal, with a whorl of phialides at the top, followed by short paired branches in right angles, each with a terminal whorl of phialides, the distance between two neighboring branches (15.0–)16.0–25.0(–27.0) μm, base not well-defined, about 3.0–4.0 μm wide. *Phialides* lateral or terminal, ampulliform or subglobose, less lageniform, widest mostly in or below the middle, in whorls, with a curved neck, (3.0–)4.0–5.0 × 3.0–4.0 μm, l/w ratio (1.1–)1.2–1.7(–1.8). *Conidia* commonly ellipsoidal to ovoid, rarely globose, thin-walled, green, smooth, (3.5–)3.7–4.6(–4.7) × (2.5–)2.6–3.5(–3.6) μm, l/w ratio (1.1–)1.2–1.6(–1.7).

Culture characteristics: Optimum temperature for growth 25 °C. No growth at 35 °C in PDA, CMD, and SNA. Colony radius on CMD after 72 h: 45–54 mm at 25 °C, 17 mm at 30 °C, covering the plate after 5 days at 25 °C. Colonies translucent, thin, margin visible, radial, indistinctly zonate. Aerial hyphae loosely disposed, inconspicuous. Chlamydospores unobserved. No diffusing pigment noted, odor indistinct. Colony radius on PDA after 72 h: 13 mm at 30 °C, covering the plate after 3 days at 25 °C. Colonies circular, zonate distinctly, white in outermost layer, green in innermost layer. Aerial hyphae abundant, cottony, disposed in one or two concentric rings around the point of inoculation in different color. Chlamydospores unobserved. No diffusing pigment noted, odor indistinct. Colony radius on PDA after 72 h: 55–65 mm at 25 °C, 7 mm at 30 °C, covering the plate after 4 days at 25 °C. Colonies hyaline, loose, circular, radial, margin not well-defined, indistinctly zonate. Aerial hyphae inconspicuous. Conidiation formed in pustules after 2 days, pustules spreading in 1–2 irregular concentric rings, commonly hemispherical around the point of inoculation, first white, turning green after 3 days. Chlamydospores unobserved. No diffusing pigment noted, odor indistinct.

Additional specimens examined: CHINA, Yunnan province, Luliang county, N24°57′22″, E103°46′30″, 1800 m alt., from soil of tobacco rhizosphere, July 2007, Z.F. Yu, living culture YMF 1.00272, YMF 1.00351 = CGMCC 3.19084.

Notes: *Trichoderma insigne* is phylogenetically close to *T. hamatum*. Morphologically, *T. hamatum* is similar to *T. insigne* for their densely clustered and ovoid phialides [61]. However, the apical and secondary branches of conidiophores of *T. hamatum* are sterile in elongation, for *T. insigne*, these structures are abundant. In addition, the phialides of *T. insigne* are significantly shorter than those of *T. hamatum* [(3.0–)4.0–5.0 vs. (4.7–)5.2–8.5 μm].

**Figure 12:**
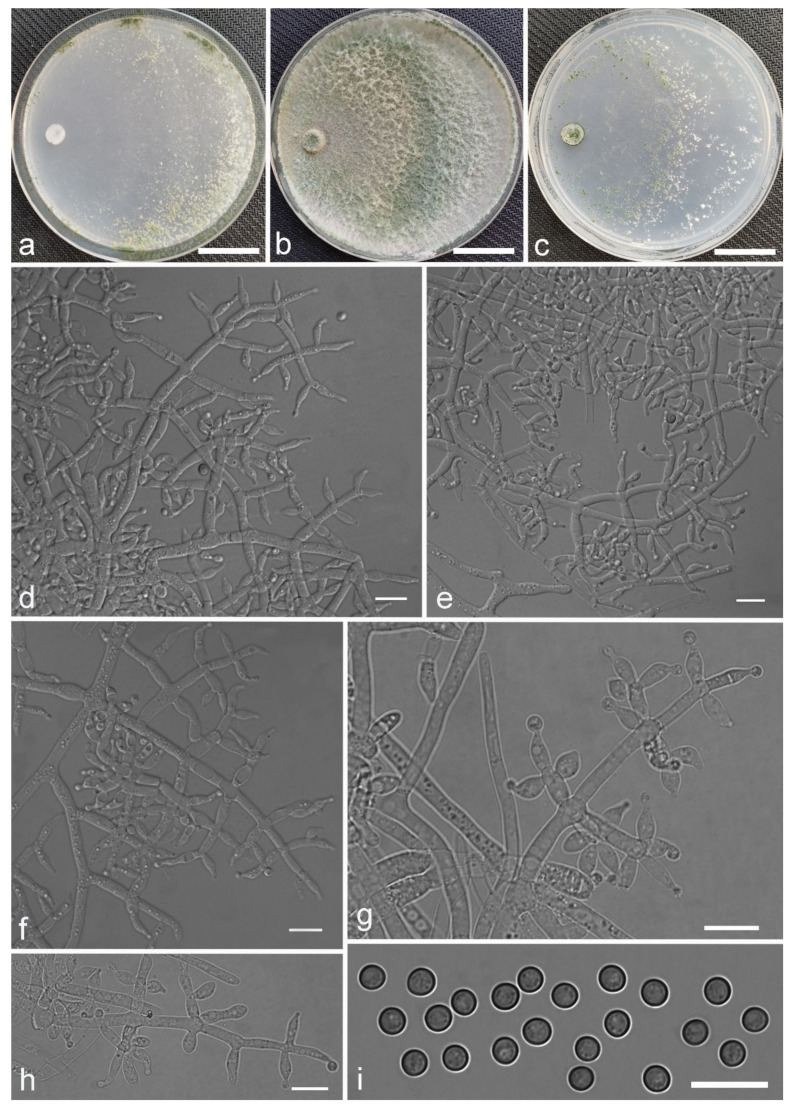
*Trichoderma obovatum* (YMF 1.06211). (**a**–**c**) Cultures after 7 d at 25 °C (**a**) on CMD; (**b**) on PDA; (**c**) on SNA). (**d**–**h**) Conidiophores, phialides and conidia formed on SNA. (**i**) Conidia. Scale bars: (**a**–**c**) = 2.5 cm, (**d**–**h**) = 10 μm.

*Trichoderma obovatum* Z.F. Yu & Y.F. Lv, sp. nov. (Figure 12)

MycoBank MB 834570

*Etymology*. Latin, *ob*-, meaning against, contrary, + *ovatum*, meaning ovoid shape, like an egg, referring to the conidia.

Type: CHINA, Yunnan province, Jianshui county, N23°20′22″, E103°06′57″, 1170 m alt., from soil, June 2018, Z.F. Yu, Y.F. Lv. Holotype YMF 1.06211, preserved in a metabolically inactive state (deep freezing) in the Conservation and Utilization of Bio-Resources in Yunnan. Ex-type culture CCTCC AF 2021069.

*Conidiophores* commonly straight, sometimes curved, emerging in right angles or oriented towards to the conidiophore axis, typically with 1–2 branched levels, side branches simple. *Phialides* ampuliform to lageniform, sometimes nearly round, mostly inhomogeneous, commonly curved or sinuous, with narrow cylindrical or hooked necks, formed mostly around the stipes at regular levels in whorls of 1–4, some growing directly on the main axis in whorls of 1–3, (4.0–)4.8–8.9(–10.7) × (2.3–)2.5–3.9(–4.2) μm, l/w ratio (1.0–)1.4–3.7(–4.3), 1.4–2.5(–3.2) μm wide at base. *Conidia* globose, oval to obovate, less commonly oblong, green, smooth, 3.2–3.8 × 3.0–3.6 μm, l/w ratio 1.0–1.2.

Culture characteristics: Optimum temperature for growth 25 °C. Colony radius on CMD after 72 h: 38–41 mm at 25 °C, 28 mm at 30 °C, covering the plate after 5 days at 25 °C, attaining 1–2 mm after 7 days at 35 °C. Colonies translucent, margin dense, center loose, inconspicuously zonate. Aerial hyphae short. Pustules firstly white, turning green after 3 days, concentrated in a marginal zone, sparse in the middle. Chlamydospores unobserved. Especially pleasant odor noted. No diffusing pigment noted. Colony radius on PDA after 72 h: cover the plate at 25 °C, 22–24 mm at 30 °C, 3–4 mm at 35 °C. Colonies grayish yellow-green to pistachio green alternately, well-defined, dense, floccose, homogeneous, indistinctly zonate. Aerial hyphae abundant, forming a dense mat. Chlamydospores unobserved. No diffusing pigment noted, odor indistinct. Colony radius on SNA after 72 h: 25–27 mm at 25 °C, 16–19 mm at 30 °C, covering the plate after 6 days at 25 °C, attaining 1–2 mm 35 °C after 7 days. Colonies hyaline, irregularly circular, indistinctly zonate. Aerial hyphae sparse, inconspicuous, mycelium creeping on the surface of media. Pustules firstly white, turning green after 3 days. Chlamydospores unobserved. No diffusing pigment noted, odor indistinct.

Additional specimen examined: CHINA, Yunnan province, Jianshui county, N23°20′22″, E103°06′57″, 2018 m alt., from soil, June 2018, Z.F. Yu, Y.F. Lv, living culture YMF 1.06212.

Notes: *Trichoderma obovatum* is characterized by its distinct odor and the rather dense pustules on CMD. Phylogenetic analyses reveal that *T. obovatum* is related to *T. paratroviride* Jaklitsch & Voglmayr, and they share the same optimal growth temperature. However, the colonies of *T. paratroviride* are slightly yellowish in reverse and chlamydospores were observed, whereas they were not observed in *T. obovatum*. Furthermore, the phialides of *T. paratroviride* are longer than those of *T. obovatum* [(5.2–)6.2–11(–14) vs. (4.0–)4.8–8.9(–10.7) um] [46].

**Figure 13:**
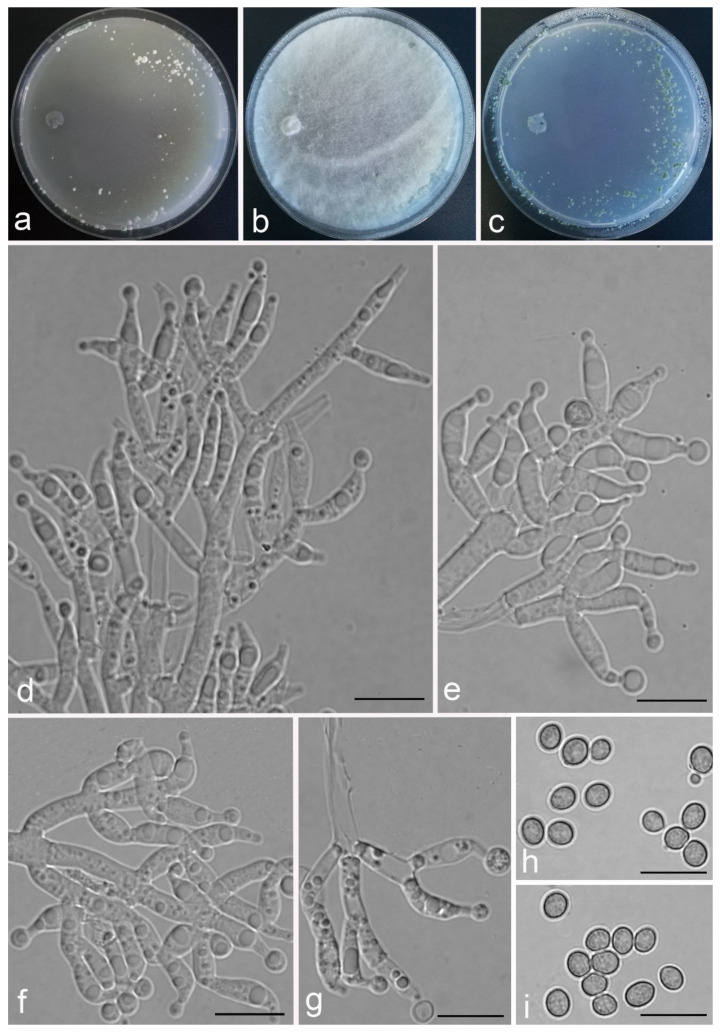
*Trichoderma paraviride* (YMF 1.04628). (**a**–**c**) Cultures after 7 d at 25 °C (**a**) on CMD; (**b**) on PDA; **c**) on SNA). (**d**–**g**) Conidiophores, phialides and conidia formed on SNA. (**h**–**i**) Conidia. Scale bars: (**a**–**c**) = 2.5 cm, (**d**–**i**) = 10 μm.

*Trichoderma paraviride* Z. F. Yu & X. Du, sp. nov. (Figure 13)

MycoBank MB830640

*Etymology.* Latin, *para*-, meaning like, resemble, -*viride*, referring to the species *Trichoderma viride*.

Type: CHINA, Sichuan province, Litang county, N30°4′12.4″, E100°7′5.1″, 4029 m alt., endophytic in living root of *Elodea nuttallii* in pond, July 2014, Z.F. Yu, Y. Huang, Holotype YMF 1.04628, preserved in a metabolically inactive state (deep freezing) in the Conservation and Utilization of Bio-Resources in Yunnan. Ex-type culture CGMCC 3.19160.

*Conidiophores* ill-defined, variably curved to sinuous extension, rarely main axis conspicuously discernible, the primary branches arising at narrow angle from the main axis, toward the tip, crowded and arranged irregularly, the secondary branches generally paired in a tree fashion, all branches terminating in a whorl of 3–5 phialides, the distance between two neighboring branches 8.6–30.1 μm, bases noted distinctly, about 2.3–3.9 um wide. *Phialides* commonly spindly to ellipsoid, rarely cylindrical, sometimes curved, the necks of phialides typically sinuous, sometimes not visible, (9.2–)9.4–15.5(–16.4) × (2.1–)2.2–3.8(–4.1) μm, l/w ratio (2.6–)2.7–5.3(–6.6). *Conidia* thin-walled, globose to subglobose, smooth, green, smooth, (3.4–)3.6–4.9(–5.0) × 3.2–4.6(–4.8) μm, l/w ratio 1.0–1.3.

Culture characteristics: Optimum temperature for growth 25 °C. No growth at 35 °C in PDA and SNA. Colony radius on CMD after 72 h: 47–51 mm at 25 °C, 43 mm at 30 °C, 4 mm at 35 °C, covering the plate after 5 days at 25 °C. Colonies hyaline, thin, margin slightly lobed, not well-defined. Aerial hyphae inconspicuous. Pustules commonly pulvinate, less hemispherical, forms relatively less, firstly white, turning green after 5 days. Chlamydospores unobserved. No diffusing pigment noted, odor indistinct. Colony radius on PDA after 72 h: 70 mm at 25 °C, 39 mm at 30 °C, covering the plate after 4 days at 25 °C. Colonies circular, white, margin wavy and surface flat. Aerial hyphae dense, continuous, arachnoid. Chlamydospores unobserved. No diffusing pigment noted. A distinct coconut-like odor noted. Colony radius on SNA after 72 h: 56 mm at 25 °C, 39 mm at 30 °C, covering the plate after 5 days at 25 °C. Colonies thin, hyaline. Aerial hyphae inconspicuous, sparse. Pustules abundant, compact, asymmetrical to hemispherical, loosely arranged in outer layer, appear within 6 days, turning green from after 7 days. Chlamydospores unobserved. No diffusing pigment noted, odor indistinct.

Notes: Phylogenetic analyses show that *Trichoderma paraviride* belongs to the *Viride* clade and is close to *T. viride* Fries. Morphologically, *T. viride* is similar to *T. paraviride* in the branching pattern of conidiophore and conidia shape; whereas the branches of *T. viride* arise from the main axis at a wide angle, the phialides of *T. viride* are lageniform to long cylindrical [46].

**Figure 14:**
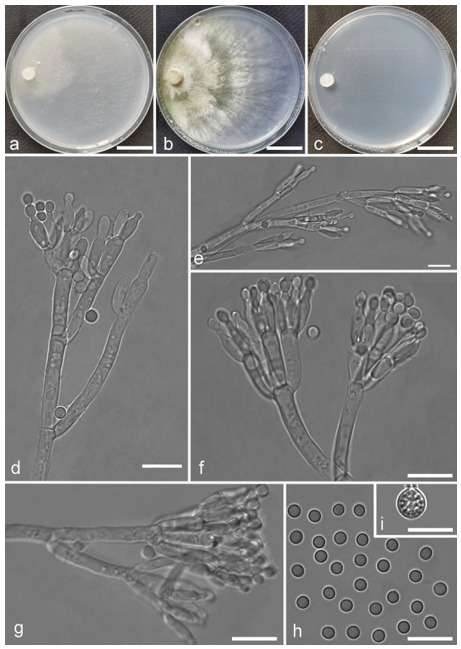
*Trichoderma pluripenicillatum* (YMF 1.06198). (**a**–**c**) Cultures after 7 d at 25 °C (**a**) on CMD; (**b**) on PDA; **c**) on SNA). (**d**–**g**) Conidiophores, phialides and conidia formed on SNA. (**h**) Conidia. (**i**) Chlamydospores. Scale bars: (**a**–**c**) = 2.5 cm, (**d**–**i**) = 10 μm.

*Trichoderma pluripenicillatum* Z.F. Yu & Y.F. Lv, sp. nov. (Figure 14)

MycoBank MB 834554

*Etymology*. Latin, *pluri*-, meaning several, many, -*penicillatum*, referring to the penicillate arrangement of the phialides.

Type: CHINA, Yunnan province, Jianshui county, N23°20′07″, E103°06′32″, 2017 m alt., from soil, June 2018, Z.F. Yu, Y.F. Lv, Holotype YMF 1.06198, preserved in a metabolically inactive state (deep freezing) in the Conservation and Utilization of Bio-Resources in Yunnan. Ex-type culture CCTCC AF 2021062. 

*Conidiophores* straight or slightly curved, *gliocladium*-like, with sparingly branching structures from the central axis, unpaired, irregular, typically asymmetrical branching of 4–6 branches in verticils in steep angles. Branches clung to the conidiophore terminus and bore ampuliform phialides that do not develop directly on the axis but aggregate at the branch terminus. Several stipes mounted on one branch terminus with a solitary phialide. *Phialides* ampuliform with symmetrical necks, (6.2–)6.5–9.0(–9.8) × 2.7–3.7(–4.0) μm, l/w ratio (1.9–)2.2–2.7(–3.1), 1.5–2.5 μm wide at the base, widest at the middle. *Conidia* oval to round, pale yellow-green, smooth, 2.7–3.4(–3.7) × (2.2–)2.5–3.2 μm, l/w ratio 1.0–1.2(–1.4). *Chlamydospores* roundness, smooth, terminal, 5.5–8.3 × 5.3–7.9 μm.

Culture characteristics: Optimum temperature for growth 30 °C. Colony radius on CMD after 72 h: 33 mm at 25 °C, 38 mm at 30 °C, 27 mm at 35 °C, covering the plate after 9 days at 30 °C. Colonies thin, translucent, indistinctly zonate, radial. Aerial hyphae scant, inconspicuous. Pustules minute, firstly white, finally turning downy light-green, surrounded the inoculation plug. A distinct odor noted. No diffusing pigment noted. Colony radius on PDA after 72 h: 36 mm at 25 °C, 42 mm at 30 °C, 35 mm at 35 °C, covering the plate after 7 days at 30 °C. Colonies zonation conspicuous, white to yellowish-white alternately, mycelium dense and radial, forming a continuous white lawn around the center and thinner at the margin. Aerial hyphae abundant, short. Yellow soluble pigments observed. A distinct odor noted. Colony radius on SNA after 72 h: 20 mm at 25 °C, 46 mm at 30 °C, 15 mm at 35 °C, covering the plate after 8 days at 30 °C. Colonies hyaline, mycelium sparse. Aerial hyphae scant, inconspicuous. No diffusing pigment noted, odor indistinct.

Notes: *Trichoderma pluripenicillatum* is located in the *Longibrachiatum* clade and distinguishable by the *gliocladium*-like conidiophores, which are uncommon in *Trichoderma*. Morphologically, *T. pluripenicillatum* is similar to *T. gliocladium* Jaklitsch & Voglmayr in the branch patterns of conidiophore and shapes of phialide, but differs in its conidia and colony characteristics: *T. pluripenicillatum* formed bright yellow pigments on PDA, while *T. gliocladium* produced orange-brown pigments [46]. Phylogenetic analyses have shown that *T. pluripenicillatum* is closely related to *T. citrinoviride* Bissett with relatively high supports, but the latter has optimal growth at 35 °C on all media, while *T. pluripenicillatum* grow better at 30 °C. Moreover, *T. citrinoviride* has shorter phialides than *T. pluripenicillatum* [(3.7–)4.7–7.8(–10.5) vs. (6.2–)6.5–9.0(–9.8) μm]. For the conidia shape, *T. citrinoviride* produces oblong or ellipsoidal conidia, but *T. pluripenicillatum* forms oval to round conidia [63].

**Figure 15:**
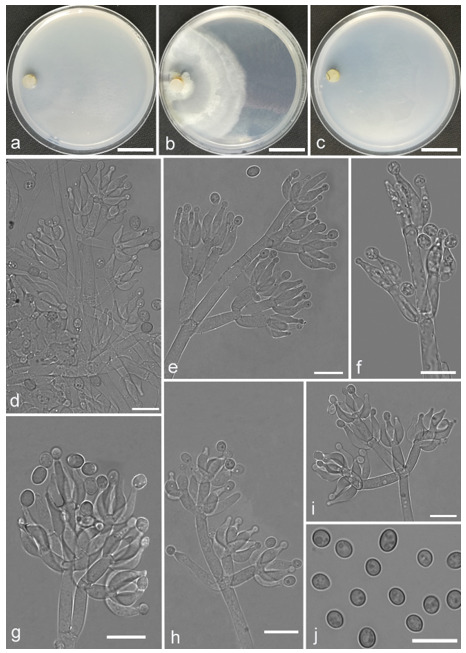
*Trichoderma propepolypori* (YMF 1.06224). (**a**–**c**) Cultures after 7 d at 25 °C (**a**) on CMD; (**b**) on PDA; (**c**) on SNA). (**d**–**i**) Conidiophores, phialides and conidia formed on SNA. (**j**) Conidia. Scale bars: (**a**–**c**) = 2.5 cm, (**d**–**j**) = 10 μm.

*Trichoderma propepolypori* Z.F. Yu & Y.F. Lv, sp. nov. (Figure 15)

MycoBank MB 834556

*Etymology*. Latin, *prope*-, meaning near, -*polypori*, referring to the phylogeny affinities with *Trichoderma polypori*.

Type: CHINA, Yunnan province, Jianshui county, N23°20′13″, E103°06′24″, 2017 m alt., from soil, June 2018, Z.F. Yu, Y.F. Lv. Holotype YMF 1.06224, preserved in a metabolically inactive state (deep freezing) in the Conservation and Utilization of Bio-Resources in Yunnan. Ex-type culture CCTCC AF 2021067.

*Conidiophores* straight or slightly curved, comprised developed main axis and paired branches at relatively regular intervals along the central axis, branches tended towards the conidiophore terminus in steep angles. *Phialides* ampulliform with sinuous, often constricted below the tip to form a narrow neck, less frequently solitary, often in whorls of 2–5, mostly inequilateral, equilateral only in central whorls, (6.5–)7.3–11.5(–14.1) × (2.8–)3.5–5.0 μm, l/w (1.3–)2.0–3.8(–5.0), (1.4–)1.9–3.0(–3.8) μm wide at the base, widest around the middle. *Conidia* pale yellow-green, smooth, ellipsoidal or oval, spheroidal present, (3.4–)3.7–4.2(–4.6) × 3.2–4.0 μm, l/w ratio 1.0–1.3. 

Culture characteristics: Optimum temperature for growth 30 °C on CMD and SNA, 25 °C on PDA. Colony radius on CMD after 72 h: 36 mm at 25 °C, 38 mm at 30 °C, 9 mm at 35 °C, covering the plate after 4 days at 30 °C. Colonies translucent, thin, flat. Aerial hyphae nearly lacking. Chlamydospores unobserved. No diffusing pigment noted, odor indistinct. Colony radius on PDA after 72 h: 55 mm at 25 °C, 32 mm at 30 °C, 9–13 mm at 35 °C, covering the plate after 4 days at 25 °C. Colonies pale white, thick, fluffy, zonate indistinctly. Aerial hyphae dense, cotton-like. Chlamydospores unobserved. No diffusing pigment noted, odor indistinct. Colony radius on SNA after 72 h: 31 mm at 25 °C, 42 mm at 30 °C, 8 mm at 35 °C, covering the plate after 4 days at 25 °C. Colonies similar to that on CMD.

Additional specimen examined: CHINA, Yunnan province, Jianshui county, N23°20′13″, E103°06′24″, 2017 m alt., from soil, June 2018, Z.F. Yu, Y.F. Lv, living culture YMF 1.06199. 

Notes: *Trichoderma propepolypori* is distinctive by the cotton-like aerial hyphae on PDA. Phylogenetically, *T. propepolypori* is closely related to *T. polypori* and *T. longifialidicum*. However, *T. propepolypori* can be easily distinguished by slower growth rate, shape of phialides, and size of conidia. The phialides of *T. propepolypori* are sinuously ampulliform phialides, while *T. polypori* is lageniform and subulate [44]. The conidia of *T. propepolypori* are much bigger than those of *T. polypori*, (3.4–)3.7–4.2(–4.6) × 3.2–4.0 vs. 2.8–3.6(−4.2) × 2.5–3.3 μm. In addition, *T. longifialidicum* has elongated and cylindrical phialides (7–21 × 1.4–2.8 μm) and simpler conidiophore patterns [54].

**Figure 16:**
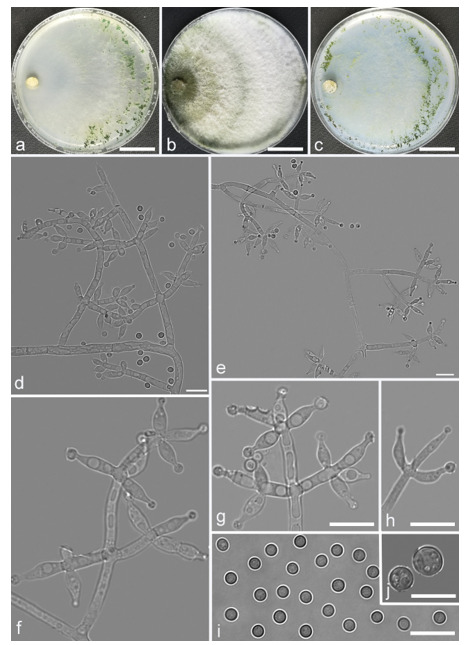
*Trichoderma pseudoasiaticum* (YMF 1.06200). (**a**–**c**) Cultures after 7 d at 25 °C (**a**) on CMD; (**b**) on PDA; (**c**) on SNA). (**d**–**h**) Conidiophores, phialides and conidia formed on SNA. (**i**) Conidia. (**j**) Chlamydospores. Scale bars: (**a**–**c**) = 2.5 cm, (**d**–**j**) = 10 μm.

*Trichoderma pseudoasiaticum* Z.F. Yu & Y.F. Lv, sp. nov. (Figure 16)

MycoBank MB 834559

*Etymology*. Latin, *pseudo*-, meaning like, similar, -*asiaticum*, referring to the species *Trichoderma asiaticum*.

Type: CHINA, Yunnan province, Jianshui county, N23°19′22″, E103°06′13″, 2010 m alt., from soil, June 2018, Z.F. Yu, Y.F. Lv. Holotype YMF 1.06200, preserved in a metabolically inactive state (deep freezing) in the Conservation and Utilization of Bio-Resources in Yunnan. Ex-type culture CCTCC AF 2021061.

*Conidiophores verticillium*-like, straight or curved, mostly emerging in aerial hyphae, typically with 1–3 branching levels, branches orientating slightly towards the conidiophore terminus, the main branches often unpaired or irregular, the side branches simple, rebranching 1–2 times, replaced by a solitary phialide or paired phialides. *Phialides* ampulliform, sometimes with narrow cylindrical or slightly bent neck, solitary or in whorls of 2–4, symmetric or inequilateral, (5.2–)6.1–9.0(–9.7) × (2.1–)2.6–3.6(–4.0) μm, l/w ratio (1.1–)1.5–3.6(–5.2), (1.0–)1.4–2.3(–2.6) μm wide at base. *Conidia* green, smooth, globose or subglobose, 2.4–3.2 × 2.4–3.0 μm, l/w ratio 1.0–1.1. *Chlamydospores* globose, relatively common, smooth, terminal, 4.7–7.7 × 4.0–7.6 μm. 

Culture characteristics: Optimum temperature for growth 25 °C. Colony radius on CMD after 72 h: 60 mm at 25 °C, 49 mm at 30 °C, 21 mm at 35 °C, covering the plate after 4 days at 25 °C. Colonies pale green, thin, circular, zonate indistinctly, radial, mycelium loose. Aerial hyphae loose, mostly concentrated on the margin. Conidiation formed in pustules after 3 days, pustules dense, firstly white, finally turning dark green. No diffusing pigment noted, odor indistinct. Colony radius on PDA after 72 h: cover the plate at 25 °C and 30 °C, 13 mm at 35 °C. Colonies white to dark green alternately, regularly circular, indistinctly zonate, radial, mycelium dense. Aerial hyphae conspicuous. No diffusing pigment noted, odor indistinct. Colony radius on SNA after 72 h: 62 mm at 25 °C, 35 mm at 30 °C, 2 mm at 35 °C, covering the plate after 4 days at 25 °C. Colonies hyaline to light green, thin, mycelium sparse. Aerial hyphae loose. Conidiation formed in pustules after 3 days, pustules green, abundant in margin. No diffusing pigment noted, odor indistinct.

Notes: *Trichoderma pseudoasiaticum* belongs to the *Harzianum* clade, and is phylogenetically close to *T. guizhouense* Q.R. Li, McKenzie & Yong Wang. However, *T. pseudoasiaticum* is obviously different from *T. guizhouense* in culture characteristics on three media and phialides. The colonies of *T. pseudoasiaticum* are white to light green and no diffusing pigment was observed, whereas those of *T. guizhouense* are white and produce brown diffusing pigmentation on PDA. Furthermore, the phialide of *T. pseudoasiaticum* is wider than that of *T. guizhouense* (2.6–3.6 vs. 2.0–3.0 μm) [64].

**Figure 17:**
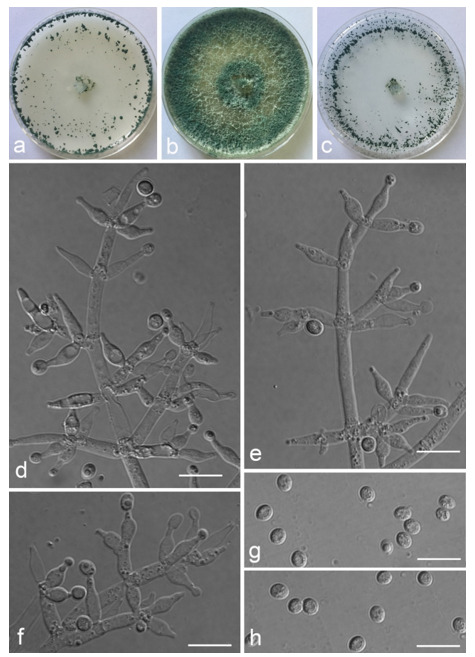
*Trichoderma pseudoasperelloides* (YMF 1.04629). (**a**–**c**) Cultures after 7 d at 25 °C (**a**) on CMD; (**b**) on PDA; (**c**) on SNA). (**d**–**f**) Conidiophores, phialides and conidia formed on SNA. (**g**–**h**) Conidia. Scale bars: (**d**–**h**) = 10 μm.

*Trichoderma pseudoasperelloides* Z.F. Yu & X. Du, sp. nov. (Figure 17)

MycoBank MB825463

*Etymology*. Latin, *pseudo*-, meaning like, similar, -*asperelloides*, referring to the species *Trichoderma asperelloides*.

Type: CHINA, Sichuan province, Xichang county, Qionghai Lake, N27°83′77.36″, E102°28′2.98″, 1520 m alt., endophytic in living leaf of *Myriophyllum spicatum*, July 2014, Z.F. Yu, Y. Huang. Holotype YMF 1.04629, preserved in a metabolically inactive state (deep freezing) in the Conservation and Utilization of Bio-Resources in Yunnan. Ex-type culture CGMCC 3.19078.

*Conidiophores* dendriform, little rebranching, often terminating in whorls of 3–5 phialides, rarely solitary, base well-defined, inconspicuous near the tip of main axis, about 3.0–5.0 um wide, branches commonly opposite, each branches terminating in a whorl of 3 or 4, the distance between two neighbor branches (14.0–)18.0–36.0(–37.0) μm. *Phialides* spindle-shaped and lageniform, (5.0–)6.0–8.0(–9.0) × (2.0–)3.0–4.0(–5.0) μm, l/w ratio (1.3–)1.4–2.7(–3.0). *Conidia* thin-walled, subglobose to ellipsoidal, rarely globose, green, smooth, (3.7–)3.8–4.0(–4.1) × (2.7–)2.8–3.3(–3.8) μm, l/w ratio (1.0–)1.1–1.4(–1.5).

Culture characteristics: Colony grows very fast on CMD and PDA. Colony radius on CMD after 72 h: cover the plate at 25 °C, 30 °C, and 35 °C. Colonies translucent, zonate indistinctly. Aerial hyphae scant, radial, inconspicuous. Pustules firstly white, gradually turning green after 3 days, relatively rare in middle, abundant at the margin. Chlamydospores unobserved. No diffusing pigment noted, odor indistinct. Colony radius on PDA after 72 h: cover the plate at 25 °C, 30 °C, and 35 °C. Colonies pale green to dark green alternately, layered distinctly, margin conspicuous, zonate indistinctly. Aerial hyphae hairy to floccose, dense, abundant in a large green disk around the inoculum. Chlamydospores unobserved. No diffusing pigment noted, odor indistinct. Colony radius on SNA after 72 h: 55–61 mm at 25 °C, 53 mm at 30 °C, 42–47 mm at 35 °C, covering the plate after 4 days at 25 °C. Colonies hyaline, circular, indistinctly zonate, mycelium sparse. Aerial hyphae sparse. Pustules dark green, relatively rare in the middle, abundant in the margin. Chlamydospores unobserved. No diffusing pigment noted, odor indistinct.

Additional specimens examined: CHINA, Sichuan province, Xichang county, Qionghai Lake, N27°83′77.36″, E102°28′2.98″, 1520 m alt., endophytic in living leaf of *Myriophyllum spicatum*. July 2014, Z.F. Yu, Y. Huang, living culture YMF 1.04633; CHINA, Yunnan province, Luliang county, N24°57′22", E103°46′30", 1800 m alt., from soil of tobacco rhizosphere, July 2007, Z.F. Yu, living culture YMF 1.00258 = CGMCC 3.19081, YMF 1.00378, YMF 1.00152. 

Notes: *Trichoderma pseudoasperelloides* is phylogenetically close to *T. asperelloides* Samuels. They are similar in shape of the phialides and conidia. But for *T. asperelloides*, secondary branches tend to be paired, also commonly unilateral and consist of a single cell near the tip of the conidiophore, which cannot be represented in *T. pseudoasperelloides* for its fascicular phialides near the tip [65]. 

**Figure 18:**
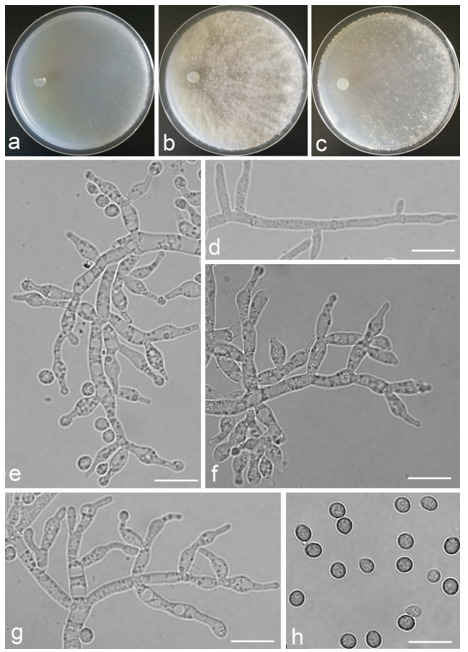
*Trichoderma scorpioideum* (YMF 1.04616). (**a**–**c**) Cultures after 7 d at 25 °C (**a**) on CMD; (**b**) on PDA; (**c**) on SNA). (**d**) Conidiophores formed on SNA. (**e**–**g**) Conidiophores, phialides and conidia formed on SNA. (**h**) Conidia. Scale bars: (**d**–**h**) = 10 μm.

*Trichoderma scorpioideum* Z.F. Yu & X. Du, sp. nov. (Figure 18)

MycoBank MB830638

*Etymology*. Latin, *scorpioideus*, referring to the scorpioid arrangement of the branches and phialides. 

Type: CHINA, Sichuan province, Daocheng county, N29°29′48.5″, E100°14′46.5″, 4362 m alt., endophytic on living stem of *Hippuris vulgaris* in pond, July 2014, Z.F. Yu, Y. Huang. Holotype YMF 1.04616, preserved in a metabolically inactive state (deep freezing) in the Conservation and Utilization of Bio-Resources in Yunnan. Ex-type culture CGMCC 3.19623.

*Conidiophores* straight or curved, comprised a slightly curved main axis and generally verticillate branches, the main axis often terminating in a whorl of 2–3 divergent phialides, the base of branches about 2.1–4.3 μm wide, branches generally toward the top of main axis and sometimes sterile, terminating 1–3 divergent phialides, the distance between two neighbor branches 12.8–18.8 μm. *Phialides* commonly narrow lageniform, some ellipsoidal, slightly swollen in the middle, the necks of phialides sometimes curved, sometimes arose singly from the main axis or branches, (6.5–)6.8–12.7(–13.3) × 2.0–3.9 μm, l/w ratio (1.8–)2.1–4.5(–4.7). *Conidia* commonly globose to subglobose, a few ovoidal to ellipsoidal, hyaline, thin-wall, green, smooth, 3.3–4.4 × 2.4–3.8 μm, l/w ratio 1.0–1.7.

Culture characteristics: Optimum temperature for growth 25 °C. No growth at 35 °C in PDA and SNA. Colony radius on CMD after 72 h: 46–54 mm at 25 °C, 32–36 mm at 30 °C, 18–20 mm at 35 °C, covering the plate after 4 days at 25 °C. Colonies translucent, thin, flat, radial, mycelium loose. Aerial hyphae sparse, inconspicuous radiate. Chlamydospores unobserved. No diffusing pigment noted, odor indistinct. Colony radius on PDA after 72 h: 55–61 mm at 25 °C, 31–37 mm at 30 °C, covering the plate after 4 days at 25 °C. Colonies white, inconspicuous radial, mycelium dense. Aerial hyphae numerous, long and wooly. Chlamydospores unobserved. No diffusing pigment noted, odor indistinct. Colony radius on SNA after 72 h: 48–53 mm at 25 °C, 27–32 mm at 30 °C, covering the plate after 4 days at 25 °C. Colonies translucent, thin, radial. Aerial hyphae sparse, arachnoid, arranged in a scattered fashion. Pustules white, minute, abundant in the margin. Chlamydospores unobserved. No diffusing pigment noted, odor indistinct.

Notes: *Trichoderma scorpioideum* is phylogenetically closely related to two species: *T. viridescens* and *T*. *sempervirentis* Jaklitsch & Voglmayr, the branches of *T. scorpioideum* and *T. viridescens* are slightly curved, whereas the branches of *T. viridescens* is paired and often terminated in 1 or 2 phialides [59], which distinctly differs from verticillate branches of *T. scorpioideum*. As for the phialides of *T. viridescens*, it often forms a submoniliform chain of 2–6 cells when cultured on CMD [59], in contrast, the structure is inconspicuous on the phialides of *T. scorpioideum*. Furthermore, the conidia of *T. viridescens* sometimes are wider (3.0–3.7 μm) than those of *T. scorpioideum*.

**Figure 19:**
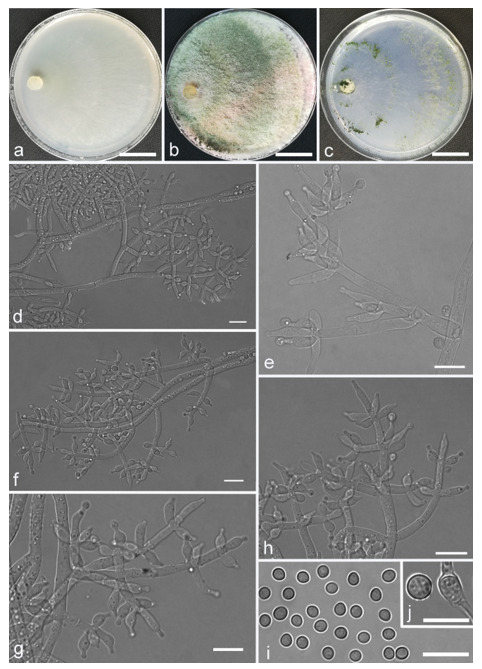
*Trichoderma simile* (YMF 1.06201). (**a**–**c**) Cultures after 7 d at 25 °C (**a**) on CMD; (**b**) on PDA; (**c**) on SNA). (**d**–**h**) Conidiophores, phialides and conidia formed on SNA. (**i**) Conidia. (**j**) Chlamydospores. Scale bars: (**a**–**c**) = 2.5 cm, (**d**–**j**) = 10 μm.

*Trichoderma simile* Z.F. Yu & Y.F. Lv, sp. nov. (Figure 19)

MycoBank MB 834560

*Etymology*. Latin, *simile*, meaning like, resemblance, which this species has resemblance to *Trichoderma guizhouense*.

Type: CHINA, Yunnan province, Jianshui county, N23°17′24″, E103°06′32″, 2015 m alt., from soil, June 2018, Z.F. Yu, Y.F. Lv. Holotype YMF 1.06201, preserved in a metabolically inactive state (deep freezing) in the Conservation and Utilization of Bio-Resources in Yunnan. Ex-type culture CCTCC AF 2021064.

*Conidiophores* tree-like, formed densely intricate reticulum, main axis unrecognizable, mostly curved, integrated into the reticulum, side branches arising from main axis asymmetrically, perpendicular to the axis, some slightly orientated towards the conidiophore terminally, rebranching 1–3 times. *Phialides* varied, borne in regular levels around the axis, mostly paired arrangements or in whorls of 2–5, sometimes crowded at the stipe terminus, less commonly singly, straight or curved, ampulliform, less lageniform with long, symmetrical or slightly bent necks, (3.8–)4.3–11.9(–14.3) × (2.3–)2.7–3.9 μm, l/w ratio 1.3–4.4(–5.2), 1.5–2.8(–3.6) μm wide at base, widest around the middle. *Conidia* oval, elliptic to subspheroidal, less oblong, green, smooth, 2.6–3.2 × 2.2–2.8 μm, l/w ratio 1.0–1.2. *Chlamydospores* elliptic or round, smooth, terminal and intercalary, 4.2–7.8 × 4.0–7.2 μm.

Culture characteristics: Optimum temperature for growth 25 °C. Colony radius on CMD after 72 h: 47 mm at 30 °C, 6 mm at 35 °C, cover the plate after 3 days at 25 °C. Colonies translucent, thin, radial. Aerial hyphae sparse. No diffusing pigment noted, odor indistinct. Colony radius on PDA after 72 h: 63 mm at 25 °C, 60 mm at 30 °C, 10 mm at 35 °C, covering the plate after 4 days at 25 °C. Colonies pale green to greyish green alternately, a few flesh-colored areas occurred on the medium, circular, conspicuously zonate. Aerial hyphae abundant, firstly white, alternately forming pale green to grayish green lawn. Pustules formed along the margin. No diffusing pigment noted, odor indistinct. Colony radius on SNA after 72 h: 47 mm at 25 °C, 45 mm at 30 °C, 3 mm at 35 °C, covering the plate after 4 days at 25 °C. Colonies translucent, thin, radial, conspicuously zonate. Aerial hyphae loose, sparse in the middle, relatively abundant in the margin. Conidiation formed on pustules, pustules minute, relatively abundant in the zonation regions. No diffusing pigment noted, odor indistinct.

Additional specimen examined: CHINA, Yunnan province, Jianshui county, N23°17′24″, E103°06′32″, 2015 m alt., from soil, June 2018, Z.F. Yu, Y.F. Lv, living culture YMF 1.06202.

Notes: Phylogenetically, *Trichoderma simile* is closely related to *T. guizhouense*. However, *T. simile* is distinguished from *T. guizhouense* by producing chlamydospores. Moreover, there are significant differences in size and shape of phialides and conidia, for instance, the phialides of *T. guizhouense*, (4.5–10 × 2.0–3.0 μm), are narrower than *T. simile*, (3.8–)4.3–11.9(–14.3) × (2.3–)2.7–3.9 μm, and *T. guizhouense* has globose conidia while the conidia of *T. simile* are oval [64]. 

**Figure 20:**
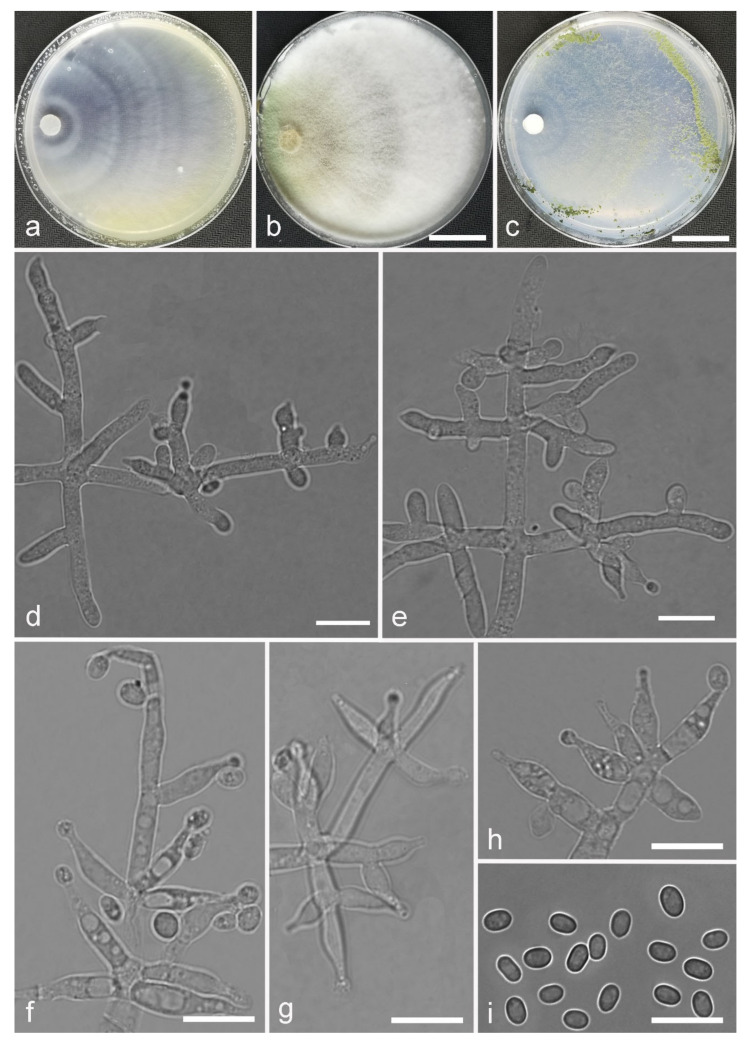
*Trichoderma subazureum* (YMF 1.06207). (**a**–**c**) Cultures after 7 d at 25 °C (**a**) on CMD; (**b**) on PDA; (**c**) on SNA). (**d**–**h**) Conidiophores, phialides and conidia formed on SNA. (**i**) Conidia. Scale bars: (**a**–**c**) = 2.5 cm, (**d**–**i**) = 10 μm.

*Trichoderma subazureum* Z.F. Yu & Y.F. Lv, sp. nov. (Figure 20)

MycoBank MB 834565

*Etymology*. Latin, *subazureum*, meaning pale bluish, referred to the color of the colonies on CMD, and SNA media.

Type: CHINA, Yunnan province, Jianshui county, N23°17′34″, E103°06′28″, 2015 m alt., from soil, June 2018, Z.F. Yu, Y.F. Lv. Holotype YMF 1.06207, preserved in a metabolically inactive state (deep freezing) in the Conservation and Utilization of Bio-Resources in Yunnan. Ex-type culture CCTCC AF 2021060.

*Conidiophores* straight or slightly curved, typically comprising 1–2 levels branched with phialides arising at the top in whorls of 2–4(–5), less commonly solitarily along stipes, with side branches short at the main axis terminus and much longer subjacent, branches paired or in whorls of 1–3, disposed perpendicular to the axis, some orientating slightly towards the conidiophore terminus. *Phialides* ampulliform to lageniform, with symmetrical or slightly curved necks, (4.7–)5.2–11.9(–20.6) × (2.2–)2.7–3.6(–4.2) μm, l/w ratio (1.3–)1.7–4.9(–8.5), (2.0–)2.2–2.9(–3.5) μm wide at base, widest around the middle. *Conidia* ellipsoidal to oblong, less globose, green, smooth, (3.2–)3.5–4.4(–4.9) × (2.1–)2.4–2.9 μm, l/w ratio (1.1–)1.4–1.8.

Culture characteristics: Optimum temperature for growth 25 °C. Colony radius on CMD after 72 h: 45 mm at 25 °C, 38 mm at 30 °C, attaining 5 mm after 7 days at 35 °C, covering the plate after 5 days at 25 °C. Colonies translucent, circular with wavy margin, zonate inconspicuously, mycelium radial, denser at the zonation rings. Aerial hyphae scant, reticular. Pustules white, minute, sparse, relatively abundant in margin. Chlamydospores unobserved. Light yellow pigments noted. No indistinct odor. Colony radius on PDA after 72 h: 60 mm at 25 °C, 47 mm at 30 °C, attaining 5 mm at 35 °C after 7 days, covering the plate after 4 days at 25 °C. Colonies white to pale bluish green, downy, thick, dense, finely wavy hyphae. Aerial hyphae abundant, dense. Chlamydospores unobserved. No diffusing pigment noted, odor indistinct. Colony radius on SNA after 72 h: 45 mm at 25 °C, 39 mm at 30 °C, attaining 3 mm at 35 °C after 7 days, covering the plate after 5 days at 25 °C. Colonies translucent to pale bluish green, fluffy, villous. Aerial hyphae dense, slightly coiling. Conidiation formed on pustules after 4 days, pustules minute, dense, relatively abundant in margin, firstly white, turning green after 7 days. Chlamydospores unobserved. No diffusing pigment noted, odor indistinct.

Notes: *Trichoderma subazureum* is characterized by crowded pustules and relatively larger conidia. Phylogenetically, it is closely related to *T. spirale* Bissett. However, *T. subazureum* is obviously different from *T. spirale* in morphology by having longer phialides, (4.7–)5.2–11.9(–20.6) vs. (3.2–) 4.5–6.5 (–8.2) μm [61]. Moreover, the growth rate of *T. spirale*, e.g. 60–70 mm on SNA after 3 d at 30 °C, is faster than that of *T. subazureum*.

**Figure 21:**
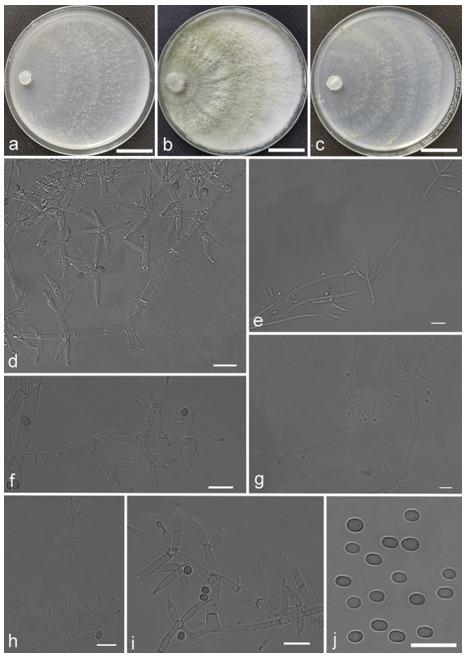
*Trichoderma subuliforme* (YMF 1.06204). (**a**–**c**) Cultures after 7 d at 25 °C (**a** on CMD; (**b**) on PDA; (**c**) on SNA). (**d**–**i**) Conidiophores, phialides and conidia formed on SNA. (**j**) Conidia. Scale bars: (**a**–**c**) = 2.5 cm, (**d**–**j**) = 10 μm.

*Trichoderma subuliforme* Z.F. Yu & Y.F. Lv, sp. nov. (Figure 21)

MycoBank MB 834564

*Etymology*. Latin*, subuliforme,* meaning subulate, awl-shaped, referring to the phialides.

Type: CHINA, Yunnan province, Jianshui county, N23°15′16″, E102°57′13″, 1170 m alt., from soil, June 2018, Z.F. Yu, Y.F. Lv. Holotype YMF 1.06204, preserved in a metabolically inactive state (deep freezing) in the Conservation and Utilization of Bio-Resources in Yunnan. Ex-type culture CCTCC AF 2021059.

*Conidiophores verticillium*-like, phialides not formed directly around the axis, side branches emerging from the main axis, perpendicular relative to the stipe axis, or orientating slightly towards the conidiophore terminus, often paired or in verticils, also solitary, mostly straight. Secondarybranches appear, no tertiary branches noted. *Phialides* subuliform, sometimes long lageniform with long neck, symmetrical or slightly curved, in whorls of 3–5(–6), (3.7–)7.0–15.8(–19.4) × (1.9–)2.4–3.0(–3.4) μm, l/w ratio (1.2–)2.4–5.9(–6.6), 1.9–3.0 μm wide at base. *Conidia* oblong to ellipsoidal, less subspheroidal, green, smooth, (2.9–)3.2–3.9(–4.3) × 2.1–2.7(–3.4) μm, l/w ratio 1.2–1.6. 

Culture characteristics: Optimum temperature for growth 25 °C. Colony radius on CMD after 72 h: 45 mm at 25 °C, 43 mm at 30 °C, attaining 23 mm at 35 °C after 7 days, covering the plate after 4 days at 25 °C. Colonies translucent, circular, loose, mycelium dense. Aerial hyphae abundant, downy. Chlamydospores unobserved. No diffusing pigment noted, odor indistinct. Colony radius on PDA after 72 h: 54 mm at 25 °C, 31 mm at 30 °C, attaining 4 mm at 35 °C after 7 days, covering the plate after 4 days at 25 °C. Colonies white to grayish yellow-green alternately, zonate inconspicuously, flocky, mycelium dense. Aerial hyphae abundant, forming a dense flat. Yellow pigments noted. No indistinct odor noted. Colony radius on SNA after 72 h: 34 mm at 25 °C, 24 mm at 30 °C, attaining 9 mm at 35 °C after 7 days, covering the plate after 6 days at 25 °C. Colonies firstly white, gradually turning grayish yellow-green, zonate, circular, mycelium dense. Aerial hyphae common. Pustule occurred on the zonate margin, firstly white, gradually turning dark green. Chlamydospores unobserved. No diffusing pigment noted, odor indistinct.

Additional specimens examined: CHINA, Yunnan province, Jianshui county, N23°15′16″, E102°57′12″, 1170 m alt., from soil, June 2018, Z.F. Yu, Y.F. Lv, living culture YMF 1.06205, YMF 1.06206.

Notes: *Trichoderma subuliforme* is phylogenetically closed to *T. spirale*. In morphology, *T. subuliforme* has slenderer phialides than *T. spirale*, (2.9–)3.2–3.9(–4.3) × 2.1–2.7(–3.4) vs. (3.6–)4.2–5.6 (−7.5) × 3.1–3.9 μm [44].

**Figure 22:**
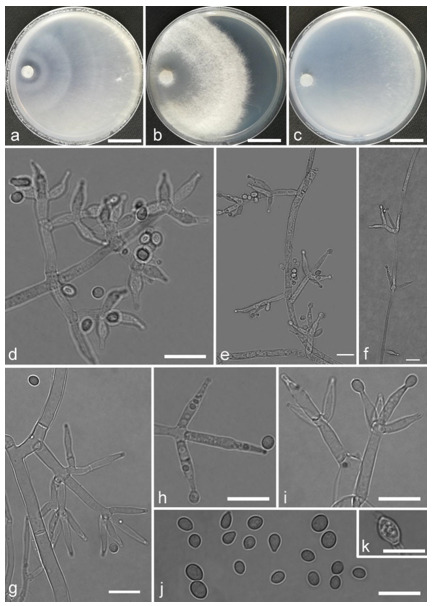
*Trichoderma supraverticillatum* (YMF 1.06208). (**a**–**c**) Cultures after 7 d at 25 °C (**a**) on CMD; (**b**) on PDA; (**c**) on SNA). (**d**–**i**) Conidiophores, phialides and conidia formed on SNA. (**j**) Conidia. (**k**) Chlamydospores. Scale bars: (**a**–**c**) = 2.5 cm, (**d**–**k**) = 10 μm.

*Trichoderma supraverticillatum* Z.F. Yu & Y.F. Lv, sp. nov. (Figure 22)

MycoBank MB 834566

*Etymology*. Latin, *supra*-, meaning above, over, -*verticillatum*, meaning verticillate, referring to the phialides. 

Type: CHINA, Yunnan province, Jianshui county, N23°15′22″, E102°57′28″, 1170 m alt., from soil, June 2018, Z.F. Yu, Y.F. Lv. Holotype YMF 1.06208, preserved in a metabolically inactive state (deep freezing) in the Conservation and Utilization of Bio-Resources in Yunnan. Ex-type culture CCTCC AF 2021063.

*Conidiophores* regularly tree-like, side branches from main axis typically paired, perpendicular to the axis at irregular intervals, some slightly orientating towards the conidiophore terminus. Stage 2 branches present, no stage 3 branches noted. *Phialides* formed mostly around the stipes at regular levels, in whorls of 3–4, paired or solitary directly on the main axis, ampuliform to spindly, homogeneous, straight, less curved or sinuous, with symmetrical or slightly bent long necks, (5.5–)7.5–11.5(–13.6) × (2.2–)2.5–3.0(–3.5) μm, l/w ratio (1.6–)2.7–4.6(–5.7), (1.9–)2.2–3.0(–3.3) μm wide at base. *Conidia* variable with two different types, ellipsoidal, less subglobose, or obpyriform to obovoid with an apparent protuberance, green, smooth, (3.2–)3.5–4.0(–4.6) × (2.3–)2.6–3.4(–3.8) μm, l/w ratio (1.1–)1.3–1.6(–1.8). *Chlamydospores* ellipsoidal, smooth, uncommon, terminal and intercalary, 5.3–8.1 × 5.0–7.9 μm.

Culture characteristics: Optimum temperature for growth 25 °C on CMD and SNA, 30 °C on PDA. Colony radius on CMD after 72 h: 49 mm at 25 °C, 43 mm at 30 °C, attaining 5 mm at 35 °C after 7 days, covering the plate after 5 days at 30 °C. Colonies translucent, circular, radial, zonate inconspicuously, mycelium scant. Aerial hyphae virtually scarce, relatively abundant at the margin, slightly coiling. No diffusing pigment noted, odor indistinct. Colony radius on PDA after 72 h: 45 mm at 25 °C, 53 mm at 30 °C, attaining 5 mm at 35 °C after 7 days, covering the plate after 5 days at 30 °C. Colonies white, circular, first hairy, later velutinous, forming a 15-mm-wide translucent ring around the inoculation plug, mycelium abundant. Aerial dense, fluffy, downy texture. No diffusing pigment noted, odor indistinct. Colony radius on SNA after 72 h: 51 mm at 25 °C, 46 mm at 30 °C, attaining 8 mm at 35 °C after 7 days, covering the plate after 5 days at 25 °C. Colonies hyaline. Aerial hyphae scant, relatively abundant at the margin. No diffusing pigment noted, odor indistinct.

Notes: *Trichoderma supraverticillatum* forms a sole clade with relatively high statistical support. It can be distinguished by two types of conidia, which are ellipsoidal or obpyriform to obovoid with an apparent protuberance. Morphologically, *T. supraverticillatum* is similar to *T. subuliforme* with characteristics of conidiophores and phialides. However, *T. subuliforme* has oblong or ellipsoid conidia, and smaller than those of *T. supraverticillatum*, (3.2–)3.5–4.0(–4.6) × (2.3–)2.6–3.4(–3.8) vs. (2.9–)3.2–3.9(–4.3) × 2.1–2.7(–3.4) μm. 

**Figure 23:**
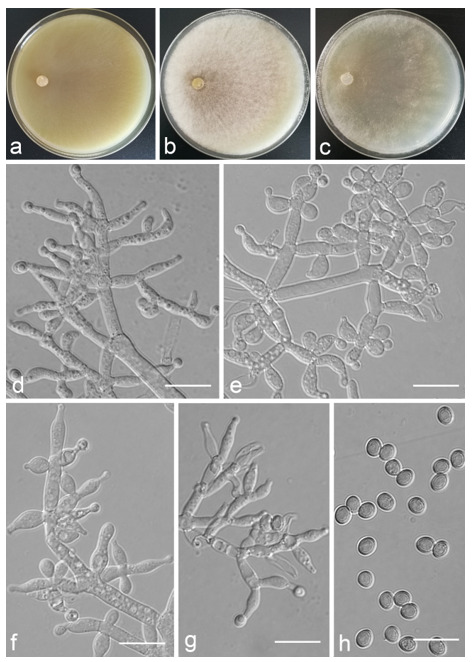
*Trichoderma tibetica* (YMF 1.05583). (**a**–**c**) Cultures after 7 d at 25 °C (**a**) on CMD; (**b**) on PDA; (**c**) on SNA). (**d**–**g**) Conidiophores, phialides and conidia formed on SNA. (**h**) Conidia. Scale bars: (**a**–**c**) = 2.5 cm, (**d**–**h**) = 10 μm.

*Trichoderma tibetica* Z.F. Yu & X. Du, sp. nov. (Figure 23)

MycoBank MB825464

*Etymology*. Latin, *tibeticum*, referring to the sampling site Tibet.

Type: CHINA, Tibet, Gongjue county, Xiongba village, N32°32′7.74″, E82°31′13.42″, 4475 m alt., endophytic on living stem of *Batrachium bungei*, August 2018, Z.F. Yu, X. Du. Holotype YMF 1.05583, preserved in a metabolically inactive state (deep freezing) in the Conservation and Utilization of Bio-Resources in Yunnan. Ex-type culture CGMCC 3.19628.

*Conidiophores* consist of a discernible, slightly curved main axis and generally paired branches, the main axis usually terminated in three cruciform phialides, the branches often slightly upward and sometimes perpendicular to main axis, the distance between two neighbor branches 10.5–34.3 μm, base slightly swollen, about 2.4–3.6 μm wide, terminating in a whorl of 2–4 divergent phialides. *Phialides* usually lageniform, sometimes globose, ellipsoidal, or pyramidal, the neck of phialides sometimes hooked or degenerated, phialides around the tip sometimes arose singly from the main axis, (5.2–)5.9–11.8(–12.3) × (2.1–)2.3–3.5 μm, l/w ratio1.9–4.5(–4.9). *Conidia* ellipsoidal to ovoid, subglobose rarely noted, green, smooth, 3.7–4.8 × (2.9–)3.1–4.0 μm, l/w ratio 1.1–1.4.

Culture characteristics: Optimum temperature for growth 25 °C. No growth at 35 °C on CMD, PDA and SNA. Colony radius on CMD after 72 h: 56–58 mm at 25 °C, 56 mm at 30 °C, covering the plate after 4 days at 30 °C. Colonies hyaline, radial, thin. Aerial hyphae sparse. Chlamydospores unobserved. Light pigments noted. No distinct odor noted. Colony radius on PDA after 72 h: cover the plate after 3 days at 25 °C and 30 °C. Colonies white, radial, flat, mycelium abundant. Aerial hyphae dense, floccose. Chlamydospores unobserved. Yellow pigments noted. No distinct odor noted. Colony radius on SNA after 72 h: 29–37 mm at 25 °C, 29 mm at 30 °C, covering the plate after 6 days at 25 °C. Colonies translucent, thin, mycelium relatively sparse around the inoculation plug. Aerial hyphae loose. Chlamydospores unobserved. Light yellow pigments noted. No distinct odor noted.

Notes: *Trichoderma tibetica* phylogenetically belongs to the *Koningii* clade, and is closely related to *T*. *petersenii* Samuels, Dodd & Schroers*. T. tibetica* and *T. petersenii* showed no significant difference in the morphological features of conidiophores, which both species having a well-defined main axis and generally paired branches, and the phialides of two species being lageniform, sometimes cylindrical, and slightly swollen in the middle [55]. But the phialides of *T. tibetica* are sometimes globose or pyramidal, and sometimes hook-like on the bent neck, which is rarely noted in *T*. *petersenii.* The conidia of *T. tibetica* are mostly ellipsoidal, sometimes ovoidal or subglobose, which distinguishes them from the generally ellipsoidal conidia of *T*. *petersenii*, and the conidia of *T*. *petersenii* are commonly smaller than those of *T. tibetica* (3.5–4.5 × 2.7–3.0 μm). In addition, no conidia formed on CMD for *T. tibetica*, only vegetative hyphae and pigment were obviously noted, but the conidia of *T*. *petersenii* appeared within 7 days on CMD [55].

**Figure 24:**
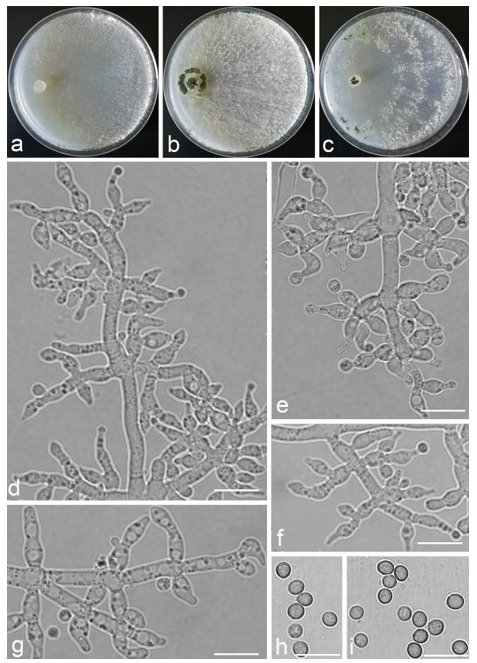
*Trichoderma uncinatum* (YMF 1.04622). (**a**–**c**) Cultures after 7 d at 25 °C (**a**) on CMD; (**b**) on PDA; (**c**) on SNA). (**d**–**g**) Conidiophores, phialides and conidia formed on SNA. (**h**–**i**) Conidia. Scale bars: (**a**–**c**) = 2.5 cm, (**d**–**i**) = 10 μm.

*Trichoderma uncinatum* Z.F. Yu & X. Du, sp. nov. (Figure 24)

MycoBank MB830641

*Etymology*. Latin, *uncinatus*, referring to the hooked, uncinated neck of the phialides.

Type: CHINA, Guizhou province, Huaxi county, N26°26′31.96″, E106°40′27.15″, 1081 m alt., endophytic on living stem of *Potamogeton malaianus* in wetland, July 2014, Z.F. Yu, Y. Huang. Holotype YMF 1.04622, preserved in a metabolically inactive state (deep freezing) in the Conservation and Utilization of Bio-Resources in Yunnan. Ex-type culture CGMCC 3.19621.

*Conidiophores* comprised a hard-discernable, slightly curved main axis, irregular alternate branches, the distance between two neighbor branches of 11.2–26.2 μm, base sometimes swollen, about 2.6–4.2 μm wide, the main axis often terminating in two divergent phialides, every branch often terminating in a whorl of 2–5 phialides. *Phialides* commonly lageniform, sometimes ampulliform to subglobose, sometimes sterile, sometime the neck of phialides uncinate or constricted sharply, rarely single, (4.3–)5.2–9.3(–10.3) × 2.3–3.9 μm, l/w ratio 1.3–3.6. *Conidia* globose to subglobose, rarely ovoidal or ellipsoidal, thin-walled, green, smooth, 3.1–4.4 × 2.7–4.0 μm, l/w ratio 1.0–1.4.

Culture characteristics: Optimum temperature for growth 30 °C on CMD and SNA, 25 °C on PDA. No growth at 35 °C on CMD, PDA and SNA. Colony radius on CMD after 72 h: 55–62 mm at 25 °C, 61–68 mm at 30 °C, covering the plate after 4 days at 30 °C. Colonies white, thin, flat. Aerial hyphae relatively scant. Pustules minute, dispersedly distributed, abundant in the margin, white at first, turning green after 5 days. Chlamydospores unobserved. No diffusing pigment noted, odor indistinct. Colony radius on PDA after 72 h: 61 mm at 30 °C, covering the plate after 3 days at 25 °C. Colonies white, loose, coarse. Aerial hyphae abundant, arachnoid. Pustules minute, abundant around the inoculation plug, firstly white, gradually turning dark green. Chlamydospores unobserved. No diffusing pigment noted, odor indistinct. Colony radius on SNA after 72 h: 43–49 mm at 25 °C, covering the plate after 3 days at 30 °C. Colonies white, circular, zonate inconspicuously. Aerial hyphae abundant. Pustules minute, formed after 3 days, turning green after 5 days, mostly distributed around the inoculation plug. Chlamydospores unobserved. No diffusing pigment noted, odor indistinct.

Notes: *Trichoderma uncinatum* is close to *T. paratroviride* in the phylogenetic tree. Morphologically, they share some characters, such as spindly branches generally toward the tip, lageniform to ampulliform phialides sometimes with curved necks, and subglobose conidia [46]. However, the distance between two neighboring branches in *T. paratroviride* is generally longer, the phialides of *T. paratroviride* are commonly in whorls of 2–4 vs. in whorl of 2–5 in *T. uncinatum*, and the phialides are spindlier (6.2–11 × 2.5–3.2 μm) than in *T. uncinatum*.

## 4. Discussion

China is considered an important reservoir of Asian biodiversity; it is estimated that this area harbours an inestimable diversity of fungi. The genus *Trichoderma* serves as a good example. The known species of the genus in China occupy 40% of the world records. Sixty-two new *Trichoderma* species have been reported since 2017 [29,30,32,44,66], which is evidence that China has tremendous *Trichoderma* diversity. Of these 62 species, 20 were discovered in southwest China, including 14 from soils, 4 from rotten woods, and 2 as endophytes from plants. For the past few years, soil has been an important substrate for investigating *Trichoderma* species, and studies focused on soil-inhabiting species of the genus have been carried out by different researchers around the world [44,46,63,67,68]. In fact, the number of *Trichoderma* species will continue to increase because many other habitats in China have not yet been investigated in a large scale. The biodiversity of *Trichoderma* on aboveground habitats may exceed that from the soil.

In recent years, we have been investigating the fungal diversity in southwest China, including in soils, submerged leaves, and aquatic plants, and described many new taxa [32,35,37,69,70,71,72,73,74,75,76,77,78,79]. In our studies, the endophytic microbiota found in these studies only collected infrequent *Trichoderma* isolates, which obtained over 2000 isolates but only 28 isolates belong to the genus *Trichoderma*. Conversely, the soil samples in these study yielded 180 isolates of *Trichoderma*, with a highly diverse taxonomic range. Finally, 23 new *Trichoderma* species are described herein, combining a phylogenetic analysis and morphological features. 

The 23 new species were assigned to nine clades in *Trichoderma* based on the two-gene phylogenetic tree, which are the *Longibrachiatum*, the *Haizianum*, the *Virens*, the *Spirale*, the *Koningii*, the *Atroviride*, the *Viridescens*, the *Viride*, and the *Hamatum*, respectively. However, the new species *T. supraverticillatum* forms phylogenetically lone lineages, and is distantly related to any other clade.

Of these 23 new species, *Trichoderma pluripenicillatum* and *T. aquatica* were grouped into the *Longibrachiatum* clade with strong support values, and their morphology also showed compelling supports in this clade as revised by Samuels et al. [6], which *Trichoderma* species in this clade are typically growing well and sporulation at 35 °C, and often produce diffusing yellow pigments on PDA. *T. pluripenicillatum* and *T. aquatica* produce yellow pigments on PDA at three temperatures, and sporulate abundantly at 30–35 °C. In addition, the conidia of this clade are typically ellipsoidal to oblong and less subglobose; *T. aquatica* fits the feature. However, *T. pluripenicillatum* has morphological differences from other species of the *Longibrachiatum* clade. *T. pluripenicillatum* produces ampuliform phialides, differing from the generally lageniform phialides of the other members of the *Longibrachiatum* clade [6]. 

Six new species, *Trichoderma achlamydosporum*, *T. propepolypori*, *T. simile*, *T. anaharzianum*, *T. asiaticum*, and *T. pseudoasiaticum*, belong to the *Harzianum* clade, which was reported by Chaverri & Jaklitsch [56] and had been specialized in multifarious morphology and complicated phylogeny. Members of the *Harzianum* clade usually form diverse pustules in culture, with different conidiophore types, phialide shapes, and varied conidia [57,67]. So far, the *Harzianum* clade, which includes over 40 species, is the largest clade of the green-spored species groups. Previously, *T. polypori* was phylogenetically related to *T. velutinum* Bissett, C.P. Kubicek & Szakács, but it is closely related to the new species *T. propepolypori* in this study. *T. guizhouense* was shown to have a close relationship with *T. harzianum* in previous studies, but is now clustered with *T. simile*, which forms a separate clade nearing to *T. harzianum* and *T. anaharzianum*. Both *T. asiaticum* and *T. achlamydosporum* form a separate subclade in the *Harzianum* clade, and differ morphologically from other species in the clade. 

The new species *Trichoderma inaequilaterale* was identified as a member of the *Virens* clade, which also belongs the group of green-spored species in *Trichoderma*, based on morphology and phylogeny. Consistent with previous studies [57], *T. crassum* and *T. virens* form a separate clade in the *Virens* clade. The general characteristics of species in this clade have a rapid growth rate and *gliocladium*-like conidiophores. 

Two new species, *Trichoderma subuliforme* and *T. subazureum*, belong to the *Spirale* clade, which is newly established by Chen and Zhuang [44] to accommodate three *Trichoderma* species, *T. hunanense*, *T. longisporum* K. Chen & W.Y. Zhuang and *T. spirale*. However, the phylogenetic position of *T. spirale* was variable. Early research by Chaverri and Samuels found that *T. spirale* was closely related to *T. polysporum* Rifai in the *Polysporum* clade [57]. Later, *T. spirale* was assigned to the *Strictipile* clade and found to be closely related to *T. longipile* Bissett and *T. strictipile* Bissett in Jaklitsch’s study [67]. Jaklitsch and Voglmayr described *T. spirale* as a separate terminal branch [46]. Based on recent study by Chen and Zhuang [44], species in the *Spirale* clade share resemble characteristics: forming downy pustules, producing yellow pigments in culture, and having oblong conidia. *T. subuliforme* and *T. subazureum* also share these characteristics with other species of the clade.

The new species *T. paraviride* belongs to the *Viride* clade, which is the largest and most diverse group of the genus *Trichoderma*. Species in this clade can be isolated from diverse sources with a wide geographic distribution [80]. The *Viride* clade was originally under the name “section *Trichoderma*” including the type species of the genus, *T. viride* [81]. Samuels et al. [55] treated them in the *T. koningii* aggregate based on the combined phenotypic and molecular data, then, Jaklitsch et al. [60] disentangled the complex. Afterwards, Jaklitsch and Voglmayr renamed the clade the *Viride* clade and provided an updated comprehensive phylogenetic tree [46]. Species in this clade mostly produce *trichoderma*-, *verticillium*- or *pachybasium*-like conidiophores with paired, verticillate phialides and green conidia.

Considering the significance of *Trichoderma* species in industry, agriculture, and ecology, the diversity and taxonomy of *Trichoderma* species are being investigated and studied by more and more researchers. The taxonomy *Trichoderma* had been studied by Samuels et al. [55], Jaklitsch [63], Jaklitsch and Voglmay [46], Chen and Zhuang [44], etc. In addition, extensive molecular studies in recent years have rapidly added new species to the genus. The taxonomy of *Trichoderma* in China dates back to 1895 [82]. Over more than a century, successive findings have brought the number of known species of the genus in China up to over 100, and species of the genus are located throughout the country. For instance, these 75 wood-inhabiting species have been found in Anhui, Fujian, Guangdong, Guangxi, Guizhou, Hainan, Hebei, Henan, Hubei, Hunan, Jiangsu, Jiangxi, Jilin, Liaoning, Shandong, Sichuan, Taiwan, Yunnan, Zhejiang provinces and Tibet region in China.

Our previous studies on the endophytic diversity of fungi in aquatic plants from southwest China found that there is abundant fungal diversity in aquatic plants. However, the isolated frequency of *Trichoderma* species is very low. Previous studies on *Trichoderma* as endophytes in terrestrial plants, particularly in their original wild to semi-wild situation, showed a considerable diversity of species. Notable examples are in *Theobroma cacao* [9,68,83] and *Hevea brasiliensis* [22,84]. Therefore, we speculate that the diversity of *Trichoderma* species in terrestrial environments is more abundant than in aquatic environments, and we will continue to study *Trichoderma* species in terrestrial plants in southwest China.

In summary, it is not easy to identify *Trichoderma* species, and impossible to define or recognize a species solely based on morphology, especially when the sexual state is absent. Now, the identification of *Trichoderma* species mainly depends on morphology, including micromorphological and cultural characters, and phylogeny. More and more DNA fragments are available for *Trichoderma* species, such as ITS, *tpb*2, *tef*1, and ACT. Of these gene fragments, ACT was introduced to study the genus, and has turned out to be efficient [60]. In the future, the species concepts of *Trichoderma* may be firmly established with the application of phylogenetic analyses at genomic level. Furthermore, the information on the ecology of the *Trichoderma* species and their function is still limited because there are many unexplored areas in China and other countries. Although our study only revealed a handful of *Trichoderma* species in the southwest region of China, our knowledge of *Trichoderma* will also provide useful information for the sufficient utilization of fungal resources. Further studies are required to understand the potential diversity of *Trichoderma* in southwest China, especially extensive surveys of unexplored areas.

## Data Availability

The data presented in this study are available in Supplementary Table S1.

## Supplementary Materials

The following are available online at https://www.mdpi.com/article/10.3390/jof7060467/s1, Table S1: GenBank accession numbers of taxa used in phylogenetic analyses.
