# Supplementary material for: New Species of Trichoderma Isolated as Endophytes and Saprobes from Southwest China
Source: J Fungi (Basel). 2021 Jun 9;7(6):467. doi: 10.3390/jof7060467 (PMC8230185; doi:10.3390/jof7060467)
Supplement: Supplementary file 1 [file jof-07-00467-s001.zip › jof-1235225-supplementary.pdf]

Table S1. GenBank accession numbers of taxa used in phylogenetic analyses.

Species name	Strain number	GenBank accession number		
		ITS	*rpb2*	*tef1*
T. achlamydosporum	**YMF 1.06226***	**MN977791**	**MT052180**	**MT070156**
T. afroharzianum	CBS 466.94	KP009262	KP009150	KP008851
	T22	—	KP009145	KP008850
T. amoenum	**YMF 1.06209***	**MN977801**	**MT052192**	**MT070146**
	**YMF 1.06210**	**MN977802**	**MT070161**	**MT070145**
T. anaharzianum	**YMF 1.00241**	**MH262584**	**MH262577**	**MH236493**
	**YMF 1.00383***	**MH113931**	**MH158995**	**MH183182**
T. anisohamatum	**YMF 1.00215**	**MH262583**	**MH262576**	**MH236494**
	**YMF 1.00253**	**MH262586**	**MH262578**	**MH236495**
	**YMF 1.00333***	**MH113926**	**MH155272**	**MH177912**
T. aquatica	**YMF 1.04624**	**MH383057**	**MK775511**	**MK775506**
	**YMF 1.04625***	**MH383058**	**MK775512**	**MK775507**
T. asiaticum	**YMF 1.00168**	**MH262582**	**MH262575**	**MH236492**
	**YMF 1.00352***	**MH113930**	**MH158994**	**MH183183**
T. asperellum	CGMCC 6422	KF425754	KF425755	KF425756
	CBS 433.97=TR3*	AY380912	EU248617	AY376058
	G.J.S. 90-7	GU198317	EU338337	EU338333
T. asperelloides	G.J.S. 04-187	JN133553	JN133560	JN133571
	G.J.S. 08-87	—	GU198272	GU198241
	G.J.S. 04-116	GU198301	GU248411	GU248412
T. asymetricum	**YMF 1.06203***	**MN977795**	**MT052186**	**MT070152**
T. asymmetricum	**YMF 1.04618***	**MK795991**	**MK795987**	**MK795983**
T. atrobrunneum	T42	KX632515	KX632572	KX632629
	S3	—	KJ665241	KJ665376
T. atroviride	TRS26	KJ786751	KP009054	KJ786832
	CBS 119499=Hypo 326	FJ860726	FJ860518	FJ860611
T. aureoviride	C.P.K. 2848=Hypo 473	—	FJ860523	FJ860615
	HMAS 266607	KF923293	KF923306	KF923280
T. citrinoviride	S20	—	KJ665250	KJ665449
	S27	—	KJ665251	KJ665450
T. crassum	G.J.S.01-227=CBS 114230	—	AY481587	JN133572
T. evansii	DIS 282d	EU856294	FJ150784	EU856319
	DIS 341hi=CBS 123079*	EU883568	EU883558	EU883566
	DIS 380a	EU856295	FJ150785	EU856320
T. gamsii	S488	—	KJ665270	JN715613
	G.J.S. 04-09	DQ315459	JN133561	DQ307541
T. guizhouense	S278	—	KF134791	KF134799
	S628	—	KJ665273	KJ665511
T. hamatum	S397	—	JN715601	JN715614
	Hypo 647	—	KJ665274	KJ665513
	Hypo 648=CBS 132565	—	KJ665275	KJ665514

T. harzianum	CBS 226.95*	—	AF545549	AF534621
	TRS55	KP009211	KP009121	KP008803
	TRS94	KP009250	KP009120	KP008802
T. hispanicum	S453*	JN715595	JN715600	JN715659
T. hunanense	HMAS 248841*	KY687924	KY687980	KY688039
	HMAS 248867	KY687950	KY688005	KY688040
T. inconspicuum	**YMF 1.04623***	**MK795993**	**MK795989**	**MK795985**
T. insigne	**YMF 1.00207***	**MH113925**	**MH155271**	**MH177911**
	**YMF 1.00272**	**MH113927**	**MH155274**	**MH177913**
	**YMF 1.00351**	**MH113928**	**MH155273**	**MH183185**
T. koningiopsis	DIS 172ai=CBS 119067	DQ313138	FJ442768	DQ284972
	DIS 229d=CBS 119069	DQ313143	FJ442712	DQ284971
T. koningii	Hypo 51=CBS 119500	FJ860762	FJ860541	KC285594
	S22	—	KC285749	KC285595
	S227	—	JN715609	KC285596
T. longibrachiatum	C.P.K. 1707	—	JN182315	EU401610
	C.P.K. 744	—	JN182308	JN182276
	S328	—	JQ685883	JQ685867
T. longiphialidicum	TC668	—	MF095871	MF095879
	TC675	—	MF095872	MF095880
T. longipilis	CBS 120953	—	FJ860542	FJ860643
	CBS 135570	—	KJ665292	KJ665556
T. longisporum	HMAS 248843*	KY687926	KY687982	KY688043
	HMAS 248868	KY687951	KY688006	KY688044
T. obovatum	**YMF 1.06211***	**MN977803**	**MT038432**	**MT070144**
	**YMF 1.06212**	**MN977804**	**MT038433**	**MT070143**
T. ovalisporum	DIS 172i	DQ323438	FJ442701	DQ288999
	DIS 70a=CBS 113299*	AY380897	FJ442742	AY376037
T. paraviride	**YMF 1.04628***	**MK775514**	**MK775513**	**MK775508**
T. parareesei	CBS 125925=C.P.K. 717*	—	HM182963	GQ354353
	C.P.K. 634=TUB F-430	—	HM182968	GQ354351
T. paratroviride	S385=CBS 136489*	—	KJ665321	KJ665627
	S489	—	KJ665322	KJ665628
T. paraviridescens	CBS 119321=Hypo 372*	NR_134367	KC285763	DQ672610
	S36	—	KC285766	KC285678
T. petersenii	CBS 119507=Hypo 45	FJ860806	FJ860568	FJ860670
	S109	—	KJ665325	KJ665631
	S167	—	KJ665326	KJ665632
T. pluripenicillatum	**YMF 1.06198**	**MN977788**	**MT070160**	**MT070159**
T. polypori	HMAS 248855*	KY687938	KY687994	KY688058
	HMAS 248861	KY687944	KY688000	KY688059
T. propepolypori	**YMF 1.06224***	**MN977789**	**MT052181**	**MT070158**
	**YMF 1.06199**	**MN977790**	**MT052182**	**MT070157**
T. pseudoasiaticum	**YMF 1.06200***	**MN977792**	**MT052183**	**MT070155**

T. pseudoasperelloides	**YMF 1.00152**	**MH262581**	**MH262574**	**MH236491**
	**YMF 1.00258**	**MH113924**	**MH107255**	**MH177910**
	**YMF 1.00378**	**MH262587**	**MH262580**	**MH247183**
	**YMF 1.04629***	**MH383059**	**MK775509**	**MK775504**
	**YMF 1.04633**	**MH383060**	**MK775510**	**MK775505**
T. pseudokoningii	G.J.S. 81-300	DQ083025	HM182985	AY937429
T. reesei	G.J.S. 00-89	—	JN175548	JN175599
	G.J.S. 97-38	AJ004962	JN175552	JN175603
T. saturnisporum	ATCC 28023	X93977	JN175524	JN388897
T. scorpioideum	**YMF 1.04616***	**MK795992**	**MK795988**	**MK795984**
T. sempervirentis	CBS 133498=S599*	—	KC285755	KC285632
	S601	—	KC285756	KC285633
T. simile	**YMF 1.06201***	**MN977793**	**MT052184**	**MT070154**
	**YMF 1.06202**	**MN977794**	**MT052185**	**MT070153**
T. spirale	TRS111	KP009301	KP009182	KP008963
	S212	—	KJ665348	KJ665740
T. strictipile	C.P.K. 1601	—	FJ860594	FJ860704
T. subazureum	**YMF 1.06207***	**MN977799**	**MT052190**	**MT070148**
T. subuliforme	**YMF 1.06204***	**MN977796**	**MT052187**	**MT070151**
	**YMF 1.06205**	**MN977797**	**MT052188**	**MT070150**
	**YMF 1.06206**	**MN977798**	**MT052189**	**MT070149**
T. supraverticillatum	**YMF 1.06208***	**MN977800**	**MT052191**	**MT070147**
T. tawa	G.J.S 02-79	—	AY391955	AY392003
	G.J.S. 97-174=CBS 114233*	—	AY391956	AY392004
T. tibetica	**YMF 1.05583***	**MK779177**	**MK779178**	**MK779179**
T. tomentosum	S23	—	KJ665351	KJ665759
	S33	—	KF134793	KF134801
T. uncinatum	**YMF 1.04622***	**MK795994**	**MK795990**	**MK795986**
T. velutinum	C.P.K. 298	—	KF134794	KJ665769
T. virens	Gli39=CBS 249.59	AF099005	AF545558	AF534631
T. viride	CBS 119325	DQ677655	EU711362	DQ672615
	TRS575	KP009372	KP009081	KP008931
T. viridescens	S1	—	KC285757	KC285634
	S452	—	KC285758	KC285646
T. viridialbum	G.J.S. 07-145	—	KC285772	KC285704
	S177	—	KC285773	KC285705
	S250	—	KC285774	KC285706
Protocrea farinosa	CBS 121551	MH863119	EU703935	EU703889
Protocrea pallida	CBS 299.78	MH861137	EU703948	EU703900

Novel species introduced in this study are indicated in bold.

The type and ex-type strains are indicated with * after the strain number.
